# Ovarian recurrence risk assessment using machine learning, clinical information, and serum protein levels to predict survival in high grade ovarian cancer

**DOI:** 10.1038/s41598-023-47983-z

**Published:** 2023-11-27

**Authors:** David P. Mysona, Sharad Purohit, Katherine P. Richardson, Jessa Suhner, Bogna Brzezinska, Bunja Rungruang, Diane Hopkins, Gregory Bearden, Robert Higgins, Marian Johnson, Khaled Bin Satter, Richard McIndoe, Sharad Ghamande

**Affiliations:** 1 Center for Biotechnology and Genomic Medicine, Medical College of Georgia, Augusta University, 1120 15th Street, Augusta, GA 30912 USA; 2 Department of Obstetrics and Gynecology, Medical College of Georgia, Augusta University, 1120 15th Street, Augusta, GA 30912 USA; 3 Department of Undergraduate Health Professionals, College of Allied Health Sciences, Augusta University, 1120 15th Street, Augusta, GA 30912 USA

**Keywords:** Ovarian cancer, Cancer, Gynaecological cancer, Tumour biomarkers

## Abstract

In ovarian cancer, there is no current method to accurately predict recurrence after a complete response to chemotherapy. Here, we develop a machine learning risk score using serum proteomics for the prediction of early recurrence of ovarian cancer after initial treatment. The developed risk score was validated in an independent cohort with serum collected prospectively during the remission period. In the discovery cohort, patients scored as low-risk had a median time to recurrence (TTR) that was not reached at 10 years compared to 10.5 months (HR 4.66, p < 0.001) in high-risk patients. In the validation cohort, low-risk patients had a median TTR which was not reached compared to 4.7 months in high-risk patients (HR 4.67, p = 0.009). In advanced-stage patients with a CA125 < 10, low-risk patients had a median TTR of 68 months compared to 6 months in high-risk patients (HR 2.91, p = 0.02). The developed risk score was capable of distinguishing the duration of remission in ovarian cancer patients. This score may help guide maintenance therapy and develop innovative treatments in patients at risk at high-risk of recurrence.

## Introduction

Ovarian cancer is the most lethal gynecologic cancer in the United States and the 5th leading cause of cancer death among women^1^. The majority of women are diagnosed with advanced-stage disease of the serous histologic subtype^2^. Treatment consists of cytoreductive surgery in combination with doublet chemotherapy consisting of a platinum and taxane agent^2^. These agents are effective with over 50% of advanced-stage patients reaching remission^3^. However, 70% of women with advanced-stage disease will recur in the first 18 months and 50% will die within 5 years of their diagnosis^4,5^.

To extend survival and eliminate microscopic disease, multiple options for maintenance therapy have become available in recent years including Vascular Endothelial Growth Factor (VEGF) and poly-ADP ribose polymerase (PARP) inhibitors^6^. PARP inhibitors are very effective in patients with BRCA mutations and homologous recombination deficiency (HRD) but result in minimal improvement in progression-free survival (PFS) in patients who are homologous recombination proficient^4,7–9^. Furthermore, these medications can have significant toxicities related to bone marrow suppression and the potential to cause the development of leukemia^4,7–9^. Recent data has even shown that in certain populations PARP inhibitors may negatively impact overall survival^10^. Bevacizumab, a VEGF inhibitor, results in a mild improvement in PFS but no improvement in overall survival^5^. The ability to predict prognosis after a complete response to therapy in ovarian cancer is essential to prioritize which patients need maintenance therapy and which can be spared unnecessary toxicities.

Except for CA125, currently, there is no test or multivariate risk score available at diagnosis or remission, which can accurately predict whether a patient will be cured of their disease or rapidly recur. Furthermore, a risk score predictive of rapid recurrence, despite imaging showing no evidence of cancer, could help guide clinical trial enrollment and bench-top research to discover new therapeutics for women who will rapidly recur.

Here, we report a multicomponent risk score that can predict the recurrence of ovarian cancer in women using clinical and serum protein levels. We developed the algorithm Ovarian Recurrence risk Assessment using Clinical and serum protein LEvels (ORACLE). The ORACLE score utilizes machine learning to incorporate clinical and serum proteomic information at remission to predict the time to recurrence in a prospective cohort of high-grade ovarian cancer patients. This risk score was then validated in a second, independent, prospective cohort of high-grade ovarian cancer patients.

## Results

### Demographics

The median age of diagnosis for the discovery and validation cohort was 61.9 and 63.4 (p = 0.54). In both cohorts, greater than 75% of patients had stage 3 or 4 disease. All patients had high or moderate-grade disease. All patients were of serous, mixed serous, or undifferentiated histology. The majority (93%) of patients underwent optimal cytoreduction. For one patient in the validation cohort, this information was not available. BRCA mutation or Homologous Recombination Deficiency (HRD) status was unknown for 66% of the discovery cohort compared to 18% of the validation cohort (p < 0.001). The mean CA125 was similar between groups at 14.3 and 17.6, respectively (p = 0.498). Receipt of maintenance therapy was more common in those within the validation cohort (61%) compared to 39% in the discovery cohort (p = 0.001). The clinical and demographic data for patients is summarized in Table 1.

**Table 1:** Summary demographics for the discovery and validation cohorts.

Demographic/clinical characteristics	Discovery (n = 71)	Validation (n = 33)	p-value
Age in years, mean (SD)	61.93 (11.95)	63.45 (11.89)	0.545
Race, n (%)			
White	61 (85.9)	25 (75.8)	0.137
Black	9 (12.7)	6 (18.2)	
Other	0 (0.0)	2 (6.1)	
Unknown	1 (1.4)	0 (0.0)	
Stage, n (%)			
1	7 (9.9)	4 (12.1)	0.251
2	7 (9.9)	3 (9.1)	
3	54 (76.1)	21 (63.6)	
4	3 (4.2)	5 (15.2)	
Grade (%)			
High	69 (97.2)	33 (100.0)	0.836
Moderate	2 (2.8)	0 (0.0)	
Pathology, n (%)			
Mixed serous	3 (4.2)	1 (3.0)	0.108
Serous	68 (95.8)	30 (90.9)	
Undifferentiated	0 (0.0)	2 (6.1)	
Optimal cytoreduction, n (%)			
No	6 (8.5)	0 (0.0)	0.215
Yes	65 (91.5)	32 (100.0)	
Neoadjuvant chemotherapy, n (%)			
No	59 (83.1)	24 (72.7)	0.335
Yes	12 (16.9)	9 (27.3)	
BRCA_HRD, n (%)			
BRCA1	3 (4.2)	2 (6.1)	** < 0.001**
BRCA2	4 (5.6)	2 (6.1)	
BRCA2 VUS	1 (1.4)	0 (0.0)	
HRD	0 (0.0)	1 (3.0)	
Negative	16 (22.5)	22 (66.7)	
Unknown	47 (66.2)	6 (18.2)	
CA125	14.34 (17.56)	17.64 (27.06)	0.498
Maintenance category, n (%)			
Anti vascular	14 (19.7)	6 (18.2)	**0.001**
Anti-VEGF and PARPi	0 (0.0)	1 (3.0)	
ER antagonists	1 (1.4)	3 (9.1)	
Immunotherapy	1 (1.4)	1 (3.0)	
Kinase inhibitor	5 (7.0)	1 (3.0)	
Monoclonal antibody	1 (1.4)	0 (0.0)	
None	43 (60.6)	13 (39.4)	
PARPi alone	0 (0.0)	6 (18.2)	
PARP and immunotherapy vs. placebo*	0 (0.0)	2 (6.1)	
Taxane	6 (8.5)	0 (0.0)	

*HRD* homologous recombination deficient, *PARPi* PARP inhibitor.

Significant values are in bold.

*Two patients were part of clinical trials which have not yet unmasked whether patients have received palcebo or not.

### ORACLE risk score creation

**Figure 1:**
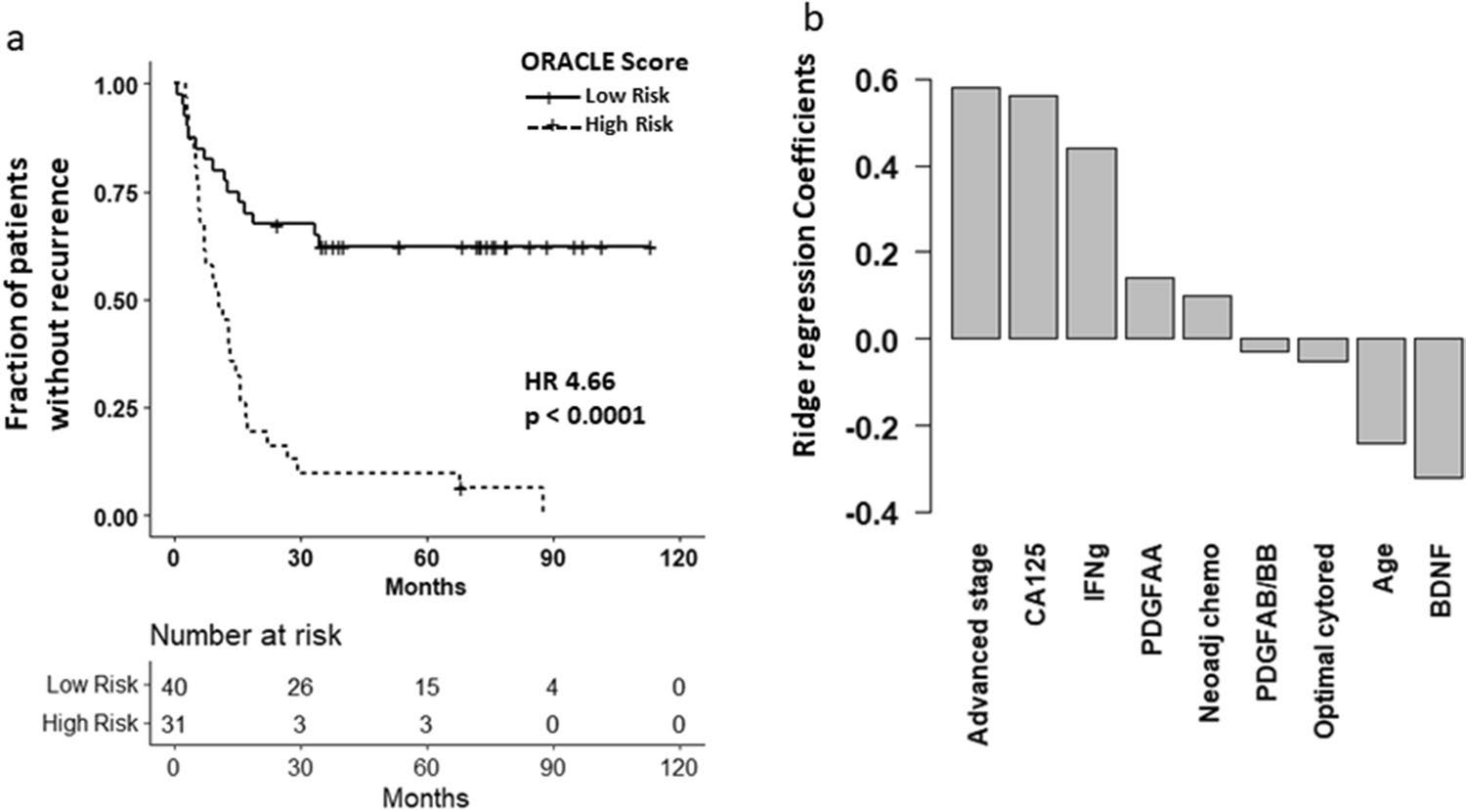
ORACLE recurrence predictions and model components. (**a**) Kaplan–Meier curve demonstrating difference in time to recurrence (TTR) between high risk and low risk patients stratified based on ORACLE Score. High risk patients had a median TTR of 10.5 months. Low risk patients had not reached their median TTR (HR 4.66, 95% CI 2.45–8.86, p < 0.0001). (**b**) Coefficients for each variable in the final model. Stage, CA125, Interferon Gamma (IFNg) and Brain Derived Neurotrophic Peptide (BDNF) were the most important predictors of TTR. Neoadj chemo: receipt of neoadjuvant chemotherapy, Optimal cytored: optimal cytoreduction.

The ORACLE score utilized the following clinical factors: patient age, optimal cytoreduction, and receipt of neoadjuvant chemotherapy, and the following serum proteins: CA125, BDNF, PDGFAA, PDGFABBB, and IFNγ (Fig. 1A). The contribution of important parameters by coefficient value for predicting patient outcome were advanced stage (0.58), CA125 (0.56), IFNγ (0.44), BDNF (− 0.32), age (− 0.24), PDGFAA (0.14), receipt of neoadjuvant chemotherapy (0.10), an optimal cytoreduction (− 0.05), and PDGFAB/BB (− 0.03) Fig. 1B. The patients were divided into two groups of low- and high-risk based on the ORACLE score (cutoff value 0.2733072).

The ORACLE score was predictive of time-to-recurrence (TTR) in the discovery data set with a concordance index of 0.72. The high-risk patients (n = 31) had a median TTR of 10.5 months compared to not reached (HR 4.66, 95% CI 2.45–8.86, p < 0.0001) after 10 years of follow up in the low-risk group (n = 40) Fig. 1A. The model was also predictive of time-to-death (TTD) with patients in the high-risk group having a median TTD of 47 months compared to not reached after 10 years of follow up in the low-risk group (HR 3.16, 95% CI 1.46–6.84, p = 0.003).

The two risk groups based on ORACLE score had similar performance when predicting PFS and OS. The median PFS for low-risk group was not reached compared to the 23-months in high-risk group (HR 4.98, 95% CI 2.60–9.53, p < 0.001). In the low-risk group the median overall survival (OS) was not reached, compared to that of high-risk group (59 months, HR 3.87, 95% CI 1.77–8.46, p < 0.001). Stage I and II patients (n = 14) were excluded from the analysis given their known good prognosis. This left 57 patients with stage 3 or 4 disease, representing advanced stage disease. ORACLE score low-risk (n = 26) with advanced stage disease had improved median PFS (85 months), TTR (34 months), OS (not reached), and TTD (72 months) compared to advanced stage, high-risk patients (n = 31) [median PFS: 23 months, HR 1.81 95% CI 1.10–2.99, p = 0.02; median TTR: 11 months, HR 3.27, 95% CI 1.67–6.41, p < 0.001; median OS: 59 months, HR 2.50, 95% CI 1.15–5.45, p = 0.02; median TTD: 47 months HR 2.09, 95% CI 0.97–4.51, p = 0.06] (Table 2).

**Table 2:** Summary of OVCAR score performance in the development and independent validation cohort.

	Development (n = 71)		Validation (n = 33)	
	Median survival	Hazard ratio and p-value	Median survival	Hazard ratio and p-value
Time to recurrence (TTR): Recurrence date − Blood sample draw date	Low-risk: Not Reached High-risk: 10.5 months	HR 4.66, 95% CI 2.45–8.86 p < 0.0001	Low-risk: Not Reached High-risk: 4.7 months	HR 4.67 CI 1.75–12.7 p = 0.002
Progression free survival (PFS): Recurrence date − Date of diagnosis	Low-risk: Not Reached High-risk: 23 months	HR 4.98, 95% CI 2.60–9.53 p < 0.001	Low-risk: Not Reached High-risk: 48 months	HR 3.71, CI 1.40–9.88 p = 0.009
Time to death (TTD): Date of death date − Blood sample draw date	Low-risk: Not Reached High-risk: 47 months	HR 3.16, CI 1.46–6.84 p = 0.003	Low-risk: 75 months High-risk: 29 months	HR 4.06, CI 1.44–11.4 p = 0.008
Overall survival (OS): Date of death − Date of diagnosis	Low-risk: Not Reached High-risk: 59 months	HR 3.87, 95% CI 1.77–8.46 p < 0.001	Low-risk: 202 months High-risk: 98 months	HR 4.58, CI 1.58–13.3 p = 0.005
Advanced stage patients				
Time to recurrence (TTR): Recurrence date − Blood sample draw date	Low-risk: 34 months High-risk: 11 months	HR 3.27, CI 1.67–6.41 p < 0.001	Low-risk: 22 months High-risk: 4.5	HR 6.29 CI 2.12–18.7 p < 0.001
Progression free survival (PFS): Recurrence date − Date of diagnosis	Low-risk: 85 months High-risk: 23 months	HR 1.81 CI 1.10–2.99 p = 0.02	Low-risk: 67 months High-risk: 39 months	HR 3.39, CI 1.23–9.36 p = 0.02
Time to death (TTD): Date of death date − Blood sample draw date	Low-risk: 72 months High-risk: 47 months	HR 2.09, CI 0.97–4.51 p = 0.06	Low-risk: 66 months High-risk: 29 months	HR 4.71, CI 1.51–14.7 p = 0.008
Overall survival (OS): Date of death − Date of diagnosis	Low-risk: Not Reached High-risk: 59 months	HR 2.50, CI 1.15–5.45 p = 0.02	Low-risk: 200 months High-risk: 98 months	HR 5.51, CI 1.64–18.6 p = 0.006

*HR* hazard ratio, *CI* 95% confidence interval.

### ORACLE validation

**Figure 2:**
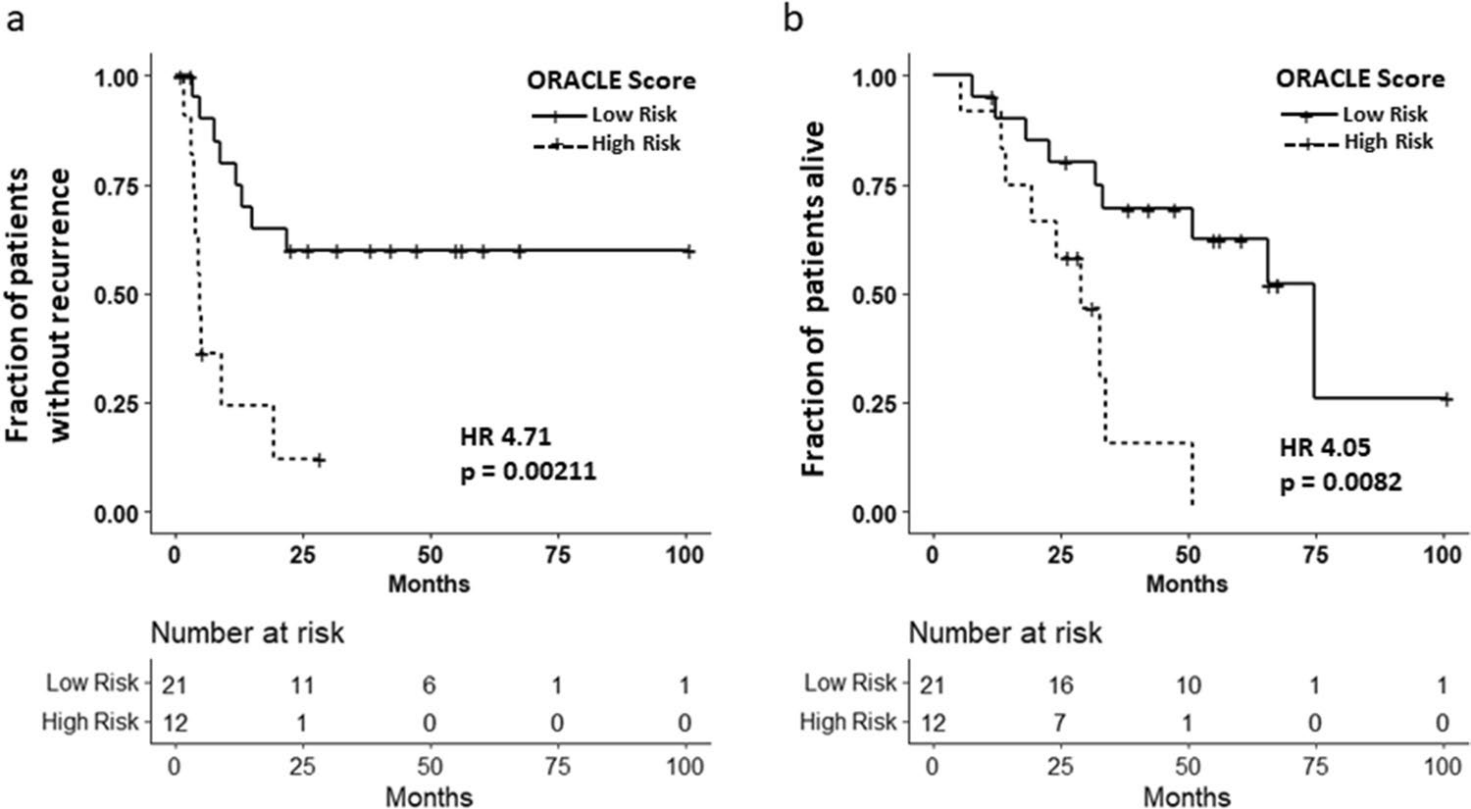
ORACLE score predicts time to recurrence (TTR) and time to death (TTD) in validation cohort. (**a**) Validation cohort Kaplan–Meier curve demonstrating difference in TTR between high-risk and low-risk patients. High-risk patients had a median TTR of 4.7 months. Low-risk patients had not reached their median TTR (HR 4.67, 95% CI 1.75–12.7, p = 0.002). (**b**) Validation cohort Kaplan Meier curve demonstrating difference in TTD between high-risk and low-risk patients. High-risk patients had a median TTD of 29 months. Low-risk patients had a median TTD of 75 months (HR 4.06, 95% CI 1.44–11.4, p = 0.008).

The ORACLE score was subsequently validated in an independent prospectively collected cohort of ovarian cancer patients at our institution (n = 33). The demographics of this cohort is described in Table 1. In this new cohort, the ORACLE score was again predictive of TTR, PFS, TTD, and OS. In the validation cohort, the median TTR for high-risk patients was 4.7 months compared to not reached in low-risk patients (HR 4.71, 95% CI 1.75–12.7, p = 0.002) (Fig. 2A). The median TTD for high-risk patients was 29 months compared to 75 months in low-risk patients (HR 4.05, 95% CI 1.44–11.4, p = 0.008) (Fig. 2B). In the validation cohort, the ORACLE was also predictive of median PFS (low: not reached, high: 48 months, HR 3.71, 95% CI 1.40–9.88, p = 0.009) and median OS (low: 202 months, high: 98 months, HR 4.58, 95% CI 1.58–13.3, p = 0.005).

When examining advanced stage patients alone (n = 26) in the validation cohort, low-risk patients had improved median PFS (67 months), TTR (22 months), OS (200 months), and TTD (66 months) compared to high-risk patients (median PFS: 39 months, HR 3.39, 95% CI 1.23–9.36, p = 0.02; median TTR: 4.5 months, HR 6.29, 95% CI 2.12–18.7, p < 0.001; median OS: 98 months, HR 5.51, 95% CI 1.64–18.6, p = 0.006; median TTD: 29 months, HR 4.71, 95% CI 1.51–14.7, p = 0.008) (Table 2). These findings validate that the ORACLE can accurately predict which patients with no evidence of disease on imaging will go on to rapidly recur and die of their disease and which will be long term survivors.

### Prognostic value of the ORACLE compared to CA125 alone

**Figure 3:**
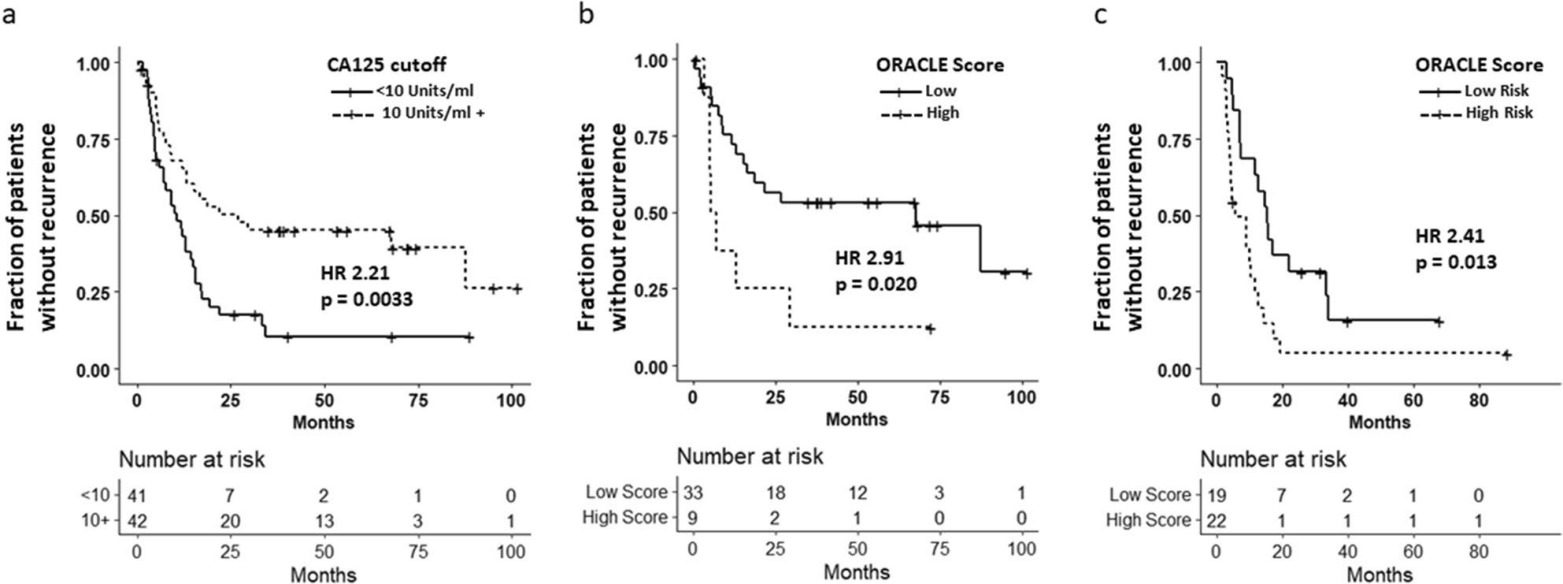
ORACLE is predictive of recurrence even in patients with low CA125 values. (**a**) Kaplan–Meier curve demonstrating difference in time to recurrence (TTR) between patients with a CA125 < 10 (low) and a CA125 > 10 (high). Those considered CA125 low had a median TTR of 27 months compared to 11 months in those that were considered CA125 high (HR 2.21, p = 0.003). (**b**) Kaplan–Meier curve comparing TTR in patients with a CA125 < 10 when divided into low and high risk ORACLE groups. The median TTR in high risk patients was 6 months compared to 68 months in ORACLE low risk patients (HR 2.91, p = 0.02). (**c**) Kaplan Meier curve comparing TTR in patients with a CA125 > 10 when divided into low and high risk ORACLE groups. The median TTR in high risk patients was 6 months compared to 16 months in ORACLE low risk patients (HR 2.41, p = 0.01).

When examining all patients with stage 3 or 4 disease who had a complete response (CR) to primary therapy, 41 patients had a CA125 ≥ 10 and 42 had a CA125 < 10. Those with a CA125 < 10 had a median TTR of 27 months compared to 11 months (HR 2.21, 95% CI 1.30–3.75, p = 0.003) in those with a CA125 ≥ 10 (Fig. 3A). To understand how the ORACLE improves prognostic prediction compared to CA125 alone, the TTR of ORACLE high and low patients was examined in patients who had a CA125 < 10 and those with a CA125 ≥ 10. Of those with a CA125 < 10 (n = 42), 33 (79%) were classified as low-risk and 9 (21%) were classified as high-risk by their ORACLE score. In this population of patients, median TTR for ORACLE low patients was 68 months compared to 6 months in ORACLE high patients (HR 2.91, p = 0.02) (Fig. 3B). In those with a CA125 ≥ 10 (n = 41), 19 patients (46%) were ORACLE low and 22 (54%) were ORACLE high. The median TTR was 16 months and 5 months (HR 2.41, p = 0.01) in ORACLE low and high groups, respectively (Fig. 3C). This data indicates that the ORACLE score provides improved prognostic prediction compared to CA125 alone in advanced staged patients.

### ORACLE score association with prognosis in BRCA mutated and BRCAwt patients

BRCA mutation status and homologous recombination deficiency (HRD) testing was available for 51 patients. Of those who underwent testing, 38 were HRD or BRCA1/2 negative (75%), 12 (24%) harbored a BRCA1/2 mutation or were HRD positive, and 1 patient (1%) had BRCA1/2 mutation which was a variant of unknown significance. Patients noted to have HRD or a BRCA1/2 mutation had a non-significant improvement in PFS (median not reached) and OS (median 202 months) compared to those who tested negative (median PFS 44 months HR 1.93, p = 0.17 and median OS 107 months; HR 1.51, p = 0.45).

When examining stratification of BRCA1/2 and HRD status by ORACLE score risk groups, 9 of the 12 patients (75%) with a BRCA1/2 mutation or HRD positivity were categorized as being in the low-risk group. The three patients who were categorized as high-risk and harbored a BRCA1/2 mutation were subsequently analyzed for outcomes. The first patient harbored a BRCA1 mutation, had a TTR of 2.8 months, and a CA125 of 9.1 at the time of her ORACLE score. The second had a BRCA1 mutation and was lost to follow up precluding analysis of TTR. However, she had a TTD of 15 months and her CA125 was 2.8 at the time of her ORACLE score. The last patient had a BRCA1 mutation, a CA125 of 8.5 at the time of her ORACLE score, and has not yet recurred after 72 months. More patients are needed to decipher if the ORACLE score can definitively predict which BRCA mutated patients are at highest risk of recurrence.

The ORACLE score was assessed for prognostic performance in BRCA and HRD negative, patients (n = 38). Those who had low ORACLE scores had a median TTR of 15 months and median TTD of 66 months, compared to a median TTR 5.0 months (HR 2.13, p = 0.054) and median TTD of 33 months (HR 2.22, p = 0.09) in high ORACLE score patients, indicating that the ORACLE score has predictive capabilities in this population of patients as well.

### ORACLE score overtime

**Figure 4:**
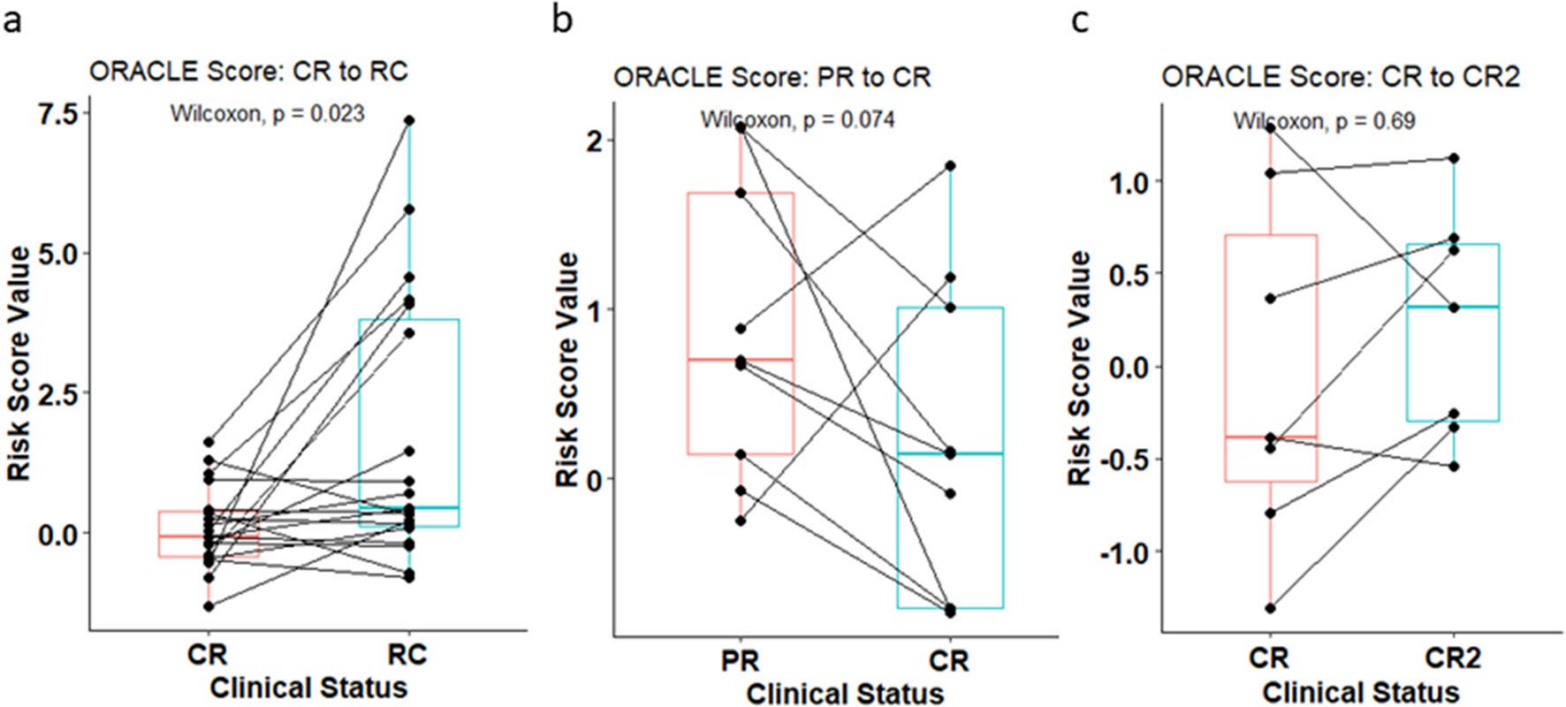
Changes in ORACLE scores during and after therapy. (**a**) Paired samples between individual patients at remission and recurrence. (**b**) Paired samples between individual patients at the time of partial response and conversion to complete response. (**c**) Paired samples between individual patients at the time of first complete response and second complete response.

Of the 104 patients, there were 19 patients with a sample at the time of complete response and at the time of their first recurrence. In this scenario, the ORACLE score elevated when patients were in the recurrent setting compared to when they were at the time of a complete response (p = 0.02) (Fig. 4A). The cohort also contained 9 patients who had a sample at the time of a partial response to their initial treatment and at the time they subsequently transitioned to a complete response. ORACLE scores of 6 out of 9 patients decreased when achieved a complete response compared to their score at the time of their partial response (p = 0.07) (Fig. 4B). Lastly, ORACLE score values were compared between the time of initial complete response and if the patient had a second complete response after treatment for recurrence (n = 7). In this scenario, the ORACLE score was similar at the time of a second complete response (p = 0.69) (Fig. 4C). The changes of the ORACLE score overtime indicates that it is a surrogate measure of ongoing activity of a patient’s cancer and could potentially be used for disease monitoring.

## Discussion

Ovarian cancer continues to be the most lethal gynecologic malignancy despite having the most new FDA approved medications over the last 10 years compared to cervical and endometrial cancer^1,11^. While most research have focused on tumor biology at diagnosis to predict recurrence and potential new treatments, limited efforts have been conducted to monitor microscopic residual disease at the time of a complete response via serum biomarkers. The ability to predict recurrence when imaging cannot identify remaining cancer is essential to begin designing treatments to eradicate the aggressive remaining microscopic disease to result in cures. The ORACLE score fills this void as the first validated machine learning risk score predictive of time to recurrence (TTR), progression free survival (PFS), time to death (TTD), and overall survival (OS) in ovarian cancer patients declared free of disease by a CT scan.

Our study was innovative in its use of machine learning to combine proteins and clinical information to predict prognosis. This score builds on prior work which has shown that machine learning can be utilized to combine multiple markers to improve patient prognostic prediction^12–15^. A major difference between our score and existing published literature is our score is applied during the remission period rather than at time of diagnosis^12–15^. We chose to focus on the remission time period since targeted therapies given during the remission time period have shown the highest success rates of extended survival in women with ovarian cancer^5,7–9^. As such, in the future the ORACLE score may be used to guide the use of maintenance therapy for women in remission. Our score also differed from existing literature scores as it combined known prognostic clinical factors such as CA125 with novel serum proteins not currently utilized to guide prognostic prediction in the clinic.

When only considering clinical factors, stage, CA125 value, receipt of neoadjuvant chemotherapy, and optimal cytoreduction were the most to least important clinical parameters. Interestingly, CA125 was a key component of a patient’s overall score, contributing approximately 20% of a patient’s total score. This is not surprising as this data and a prior randomized control trial data have shown that CA125 levels less than 10 confer improved long term prognosis^16^. However, this is not guaranteed, as patients considered ORACLE score high-risk with a CA125 value of less than 10 had a median time to recurrence of 6 months. Without the ORACLE score, this population would be expected to not recur for greater than 2 years.

Chronic low-grade inflammation is considered as one of the etiologic factors contributing to survival of neoplastic growth and malignancy in ovarian cancer^17,18^. In a previous study, women having higher serum levels of BDNF, PDGFAA and PDGFABBB, had improved PFS^19^. These three molecules are considered as important modulators of immune mediated clearance of microscopic disease after debulking surgery in ovarian cancer patients^19^. Earlier studies suggested an anti-apoptotic and tumor promoting role of BDNF, new evidence points towards promoting anti-tumor immune response and by augmenting sensitivity to chemotherapy^20^. The platelet derived molecules are shown to have both anti- and pro-tumorigenesis roles. Pro-tumorigenesis role is ascertained to stimulation of VEGF production by PDFG, which in turn promotes growth and metastasis of tumors^21^. Serum levels of PDGF can provide prognostic information has been demonstrated in various cancers including ovarian cancer^22^ after chemotherapy, radiotherapy and immunotherapy^23,24^. Clinical trials data on use of IFNγ has shown increase in OS and PFS in ovarian cancer^25^ and melanoma^26^ patients. Cytokine IFNγ produced by T-cells, is considered as a molecule with dual roles, which promotes anti-tumor immunity and immune evasion^27,28^. In this study, high serum levels of IFNγ is a strong contributor to having a higher ORACLE score, suggesting that increased levels of IFNγ are contributing to immune evasion in patients resulting in a decreased recurrence free survival^29^. In this study, it is evident that the serum proteins, BDNF, IFNγ, PDGFAA, and PDGFABBB are detecting physiologic signals predictive of underlying disease activity that CA125 is unable to detect alone.

The performance of the ORACLE score in BRCA mutated and BRCAwt/HRD negative patients is another exciting aspect of our research findings. Interestingly, 75% of BRCA mutated patients were in the low-risk group based on the ORACLE score. However, of those in the high-risk group, all three had CA125s of less than 10 and the ORACLE score correctly predicted short term prognosis of each of these patients except for one. Additional studies are needed to better understand if the ORACLE score will be able to stratify BRCA mutated patients into high and low-risk populations. This would be beneficial as it could allow low-risk patients to be spared the toxicities of PARP inhibitors^4,7–9^. In the BRCAwt and homologous recombination proficient population, the ORACLE was again able to identify patients who would live beyond 5 years and those who would have an unexpectedly short prognosis despite having imaging which indicated that no cancer was present. More patients are needed to definitively understand how the ORACLE score will predict prognosis and benefit of PARP use in those who are BRCAwt and homologous recombination proficient population. Despite the number of strengths of our prospective study, there are limitations which need to be addressed before moving the ORACLE score to routine clinical use.

One limitation of our data is that it is a single site study and the proportion of patients without BRCA testing. In the discovery data set, only 34% of patients had undergone BRCA testing. However, this is not surprising as the median year of diagnosis was 2010 which is the same year universal BRCA testing was recommended in ovarian cancer^30^. However, 34% is better than expected when considering that national rates of BRCA testing for ovarian cancer in 2014 were as low as 10–30%^31^. The validation cohort had excellent rates of BRCA testing with 82% of patients having undergone testing. The low rate of BRCA testing in the discovery set precluded us from including BRCA mutation status or HRD in the algorithm. However, as stated above, it appears that the proteomic signature served as a surrogate for BRCA mutation status as the majority of these patients were classified as low-risk. In fact, with more patients, this risk score may successfully differentiate which patients with a BRCA mutation will have short versus long term survival.

Last, although these samples were collected prospectively, there were multiple limitations in regards to patient follow up and serum collection. In regards to follow up, patients often come to our institution from hours away and thus see a local oncologist for a portion of their survivorship visits. This caused some of the patients to have remission samples collected at different points in relation to therapy completion, which could potentially confound results. For this reason we analyzed both time to recurrence (time from blood draw to progression) and progression free survival (time from diagnosis to progression) for all patients. The ORACLE was predictive of both time to recurrence and progression free survival which indicates that the risk score was accurately predicting ongoing disease biology.

Despite these limitations, this represents the first validated machine learning risk score utilizing clinical and proteomic information at remission to predict prognosis in ovarian cancer patients. Furthermore, this risk score was developed as part of a prospective serum collection which reduces the potential for bias. The score warrants multi-center validation in ongoing ovarian cancer clinical trials with concurrent studies investigating how this risk correlates to tumor biology and can be used to predict maintenance therapy benefit and design new maintenance therapies capable of destroying remaining microscopic disease.

## Methods

### Study population

This was a single-institution, prospective, observational study examining serum samples in patients with serous or undifferentiated high grade ovarian cancer. The study was approved by the institutional review board at the Medical College of Georgia at Augusta University. Written informed consent was compliant with the ethics and committee at Augusta University and was obtained from all patients. All women with ovarian cancer were prospectively monitored and enrolled into Biomarkers and Therapy for Cancer Repository at our institution^19,32^. This study is compliant with all institutional and national guidelines and regulations for human subjects research.

Blood was obtained at enrollment and then at subsequent follow up visits, including during treatment, remission, and recurrence. Blood samples were collected in serum separator tubes (BD Biosciences), allowed to clot for 30 min at room temperature. Serum was obtained after centrifugation and further aliquoted into wells of 96-well plates (150 μl/well) to create master plates. Daughter plates were then created by pipetting 5–25 μl of serum/well to avoid repeated freeze/thaw for all samples. Samples were aliquoted and stored in a − 80 °C freezer until use. The first available sample from each clinical time point for each patient was used for analyses.

All patients were treated with cytoreductive surgery and doublet chemotherapy with a platinum and taxane agent. Optimal cytoreduction was defined as < 1 cm of residual disease at the time of cytoreductive surgery. Patients were not excluded for having received neoadjuvant chemotherapy, maintenance therapy, or being on a clinical trial, as long as they received a platinum and taxane agent for initial therapy (Table 1).

A complete and partial response to primary therapy was defined in accordance to Revised RECIST criteria (version 1.1) in combination with physical exam, clinical imaging, and CA125 after at least four cycles of initial platinum and taxane therapy^33^. No response to primary therapy was defined as progression of disease on imaging in accordance to Revised RECIST criteria (version 1.1) after four cycles of initial platinum and taxane therapy. A recurrence was defined as the time a patient met criteria for progression base on RECIST (version 1.1) in combination with physical exam, clinical imaging, and CA125 after having a complete response to primary therapy^33^. Patient records were reviewed for clinical data including demographics, pathologic characteristics, treatment information, molecular data, time of recurrence, and time of death. A CA125 value of 10 was used to divide patients into high and low CA125 value groups based on data from the clinical trial GOG252 which showed that this cutoff was associated with identification of long term survivors with advanced stage ovarian cancer (Table 1)^16^.

### Patient population for ORACLE development and validation

The discovery cohort included a total of 71 patients who reached remission with serous high grade ovarian. The median year of diagnosis for this cohort was 2010. BRCA testing was not performed on tumor specimens of those who had died given the potential ethical implications of a positive result for their family members.

The validation cohort consisted of 33 new patients who reached remission with high grade serous or undifferentiated ovarian cancer. The median year of diagnosis for these patients was 2015. The first available remission sample for patients in both the discovery and validation cohort was chosen for analysis. Subject demographics are described in the results section.

### Luminex assays

In prior work, 26 proteins were analyzed for their association with survival^19^. Of these, Brain Derived Neurotrophic Factor (BDNF), Platelet Derived Growth Factor AA (PDGFAA), Platelet Derived Growth Factor ABBB (PDGF ABBB), and Interferon Gamma (IFNγ) were the most promising and thus were measured in the validation cohort^19^.

Luminex assays for the above mentioned proteins were obtained from Millipore (Millipore Inc., Billerica, MA, USA). Assays were performed utilizing our previously described methods^19^. For reproducibility, these methods are repeated here: Luminex assays were performed according to instructions provided with the kit. Serum samples were incubated with capture antibodies immobilized on dye-encoded polystyrene beads for 1 h. The beads were then washed and further incubated with biotinylated detection antibody cocktail for 1 h. Next, phycoerythrin-labeled streptavidin was added to the wells and incubated for 30 min. The beads were washed for final time and suspended in 60 μl of wash buffer. The median fluorescence intensities (MFI) were measured using a FlexMAP 3D array reader (Millipore, Billerica, MA) with the following instrument settings: events/bead: 50, minimum events: 0, flow rate: 60 μl/min, Sample size: 50ul and discriminator gate: 8000–13,500. Before performing the profiling, assays were performed at different serum dilutions to ensure the MFI values of the samples were within the linear range of the standard curve. Luminex median fluorescence intensity (MFI) data was subjected to quality control steps as described in our earlier study^34^. In brief, The MFI data for replicate wells was also checked and wells with coefficient of variation (CV) > 25% were not included in further analyses. Wells with low bead counts (below 30), or high bead CV (above 200) were flagged for exclusion. Protein concentrations were estimated using a regression fit to the standard curve with known concentration included on each plate using a serial dilution series. To achieve normal distribution, MFI and concentrations for standards were log2 transformed prior to all statistical analyses.

### Harmonization of proteomic data

To control for batch effects between the discovery and validation cohort, BDNF, PDGFAA, PDGFABBB, and IFNγ levels were harmonized between the discovery cohort of patients and the validation set. This was done via the empirical Bayes method using the Surrogate Variable Analysis package in R^35,36^. Visualization of the proteomic data after controlling for batch effects can be seen in Supplementary Fig. 1. After harmonization, the proteins BDNF, PDGFAA, and PDGFABBB, were reassessed for their association with survival in the discovery data set to confirm that controlling for batch effects did not alter the survival Supplementary Table 1^19^.

### Creation of the ORACLE score

In order to create the Ovarian Recurrence risk Assessment using Clinical and serum protein LEvels (ORACLE) score that accounted for all clinical and serum data, we used the elastic net algorithm to combine clinical, pathologic and proteomic patient data^37^. Specifically, the elastic net algorithm combined patient age at diagnosis, receipt of neoadjuvant chemotherapy, an optimal cytoreduction, advanced stage, CA125 value, BDNF level, IFNγ level, PDGFAA level, and PDGFABBB level into a single risk score. Advanced stage was considered as stage 3 or 4 disease. This was coded as a 1 for those who had advanced stage and 0 if they did not have advanced stage. There were 18 of 104 patients who had missing values for CA125. These missing values were imputed using the caret package in R because the elastic net algorithm is unable to handle missing values^38^. All data was centered and scaled prior to application of the algorithm.

The elastic net algorithm combines the multiple predictors in a linear combination and tunes the model base on a penalty term, which is the sum of the square of the coefficients used in the model. The effect of the penalty term can be adjusted to either have no effect lambda = 0 or as lambda approaches infinity, variable coefficients approach 0. The optimum lambda was determined using the lambda.min function in R, which automatically chooses the best lambda value to eliminate errors on cross validation. The sum of the linear combination yields a composite score for each individual patient. The number of predictors is further optimized by varying an alpha value from 0 to 1. Where an alpha of 0 includes all possible predictors, while an alpha of 1, decreases the number of predictors to the lowest number possible. The composite score of the combined predictors for each value of alpha and lambda were then subject to survival analysis and cox proportional hazards to determine the optimum score for predicting time to recurrence (TTR). In the discovery set, 20 k-fold internal cross validation found that an alpha of 0 provided the highest concordance index which is a measure of performance for survival prediction. Models for other levels of alpha are shown in Supplementary Fig. 2. The optimal cut-off was chosen to divide patients into two different risk groups that would provide the highest hazard ratio and lowest p-value. This value was determined to be 0.2733072. The model was then saved for application to the validation cohort. The same cutoff of 0.2733072 was used to separate patients into high and low-risk groups in the validation cohort.

### Primary study objective and survival definitions

The primary objective of this study was to determine if the ORACLE score was predictive of time to recurrence in the validation cohort. Time to recurrence (TTR) was defined as the time from blood draw to recurrence. This is similar to prior studies which have examined the relationship of a rising CA125 or CEA to the time a recurrence is visible on imaging or detected clinically^39–41^. Other survival analyses included progression free survival (PFS), which was the time of diagnosis to time of first recurrence, time to death (TTD), which was the time of blood draw to date of death, and overall survival (OS) which was calculated as the time of diagnosis to time of death.

### Power analysis for validation

**Figure 5:**
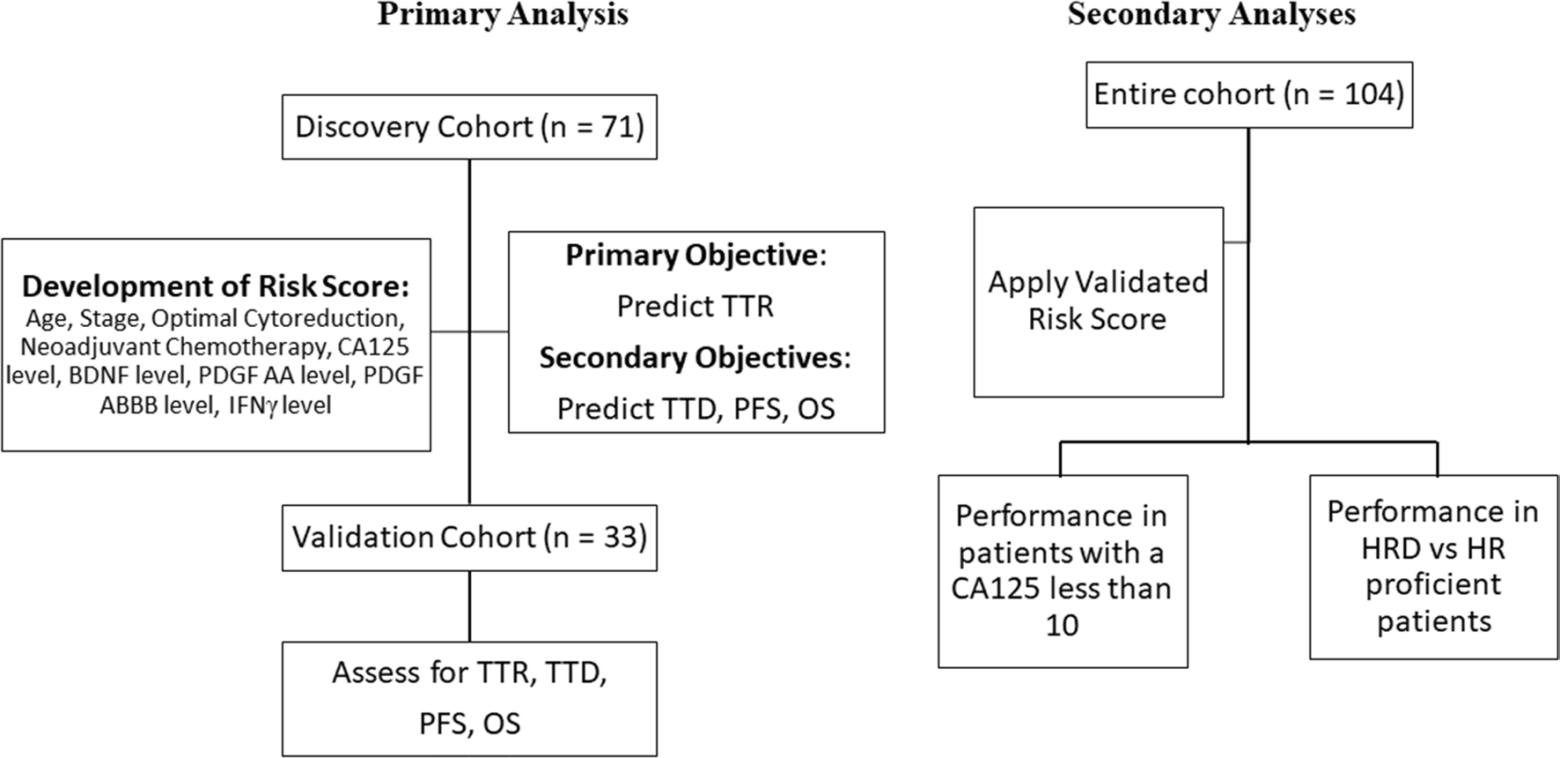
Study schema and objectives. Visualization of the planned analyses. Prospectively enrolled patients in the discovery cohort were used to develop a risk score combining clinical and molecular information. This risk score was subsequently validated in an independent cohort. Preplanned subgroup analyses related to prognostic performance of the risk score in those with a CA125 < 10 and in patients with and without homologous recombination deficiency. Time to recurrence (TTR) time from blood draw to recurrence. Progression free survival (PFS) time from diagnosis to recurrence, time to death (TTD) time of blood draw to date of death, overall survival (OS) time of diagnosis to time of death. Brain Derived Neurotrophic Factor (BDNF), Platelet Derived Growth Factor AA (PDGFAA), Platelet Derived Growth Factor ABBB (PDGF ABBB), and Interferon Gamma (IFNγ).

Based on a hazard ratio of 4.5, 33 patients with 13 progression events and 16 deaths would provide 80% powerto validate the TTR and TTD prediction. At the time of this analysis there were 17 progression events and 18 deaths which provided a power of 99.6% for TTR and 94.3% for TTD. A Consort Diagram of patients and the planned analyses are shown in Fig. 5.

### Statistical analysis

All statistical analyses were performed using the R language and environment for statistical computing (RStudio version 4.2.0; R Foundation for Statistical Computing; www.r-project.org). The statistical significance of differences was set at p < 0.05, all p values were two sided. Continuous variables were reported as mean ± standard deviation and categorical variables as numbers and percentages. Differences between groups were analyzed by *Chi-squared* test for categorical variables and Student’s *t*-test test for continuous variables. Cox proportional hazards models were used to evaluate the impact of clinical factors and serum protein levels on survival. These results are reported with corresponding 95% confidence intervals. Differences in survival (OS and PFS) are shown as Kaplan–Meier plots. Patients with no history of recurrence or death were censored at the date of last follow-up visit. Patients who died of natural causes unrelated to cancer were censored at time of death. Kaplan–Meier survival analysis and log-rank test were used to compare differences in survival.

### Supplementary Information

Supplementary Figure 1.

Supplementary Figure 2.

Supplementary Table 1.

## Data Availability

Please contact Dr. David Mysona at dmysona@augusta.edu for data requests. All requests will be granted as expeditiously as possible.
