# Supplementary material for: Ovarian recurrence risk assessment using machine learning, clinical information, and serum protein levels to predict survival in high grade ovarian cancer
Source: Sci Rep. 2023 Nov 27;13:20933. doi: 10.1038/s41598-023-47983-z (PMC10684567; doi:10.1038/s41598-023-47983-z)

Supplementary Figure 1

Supplementary Figure 1. Plot showing that after using the empirical Bayes Method both datasets overlap. PC1 and PC2 represent the validation and discovery datasets.

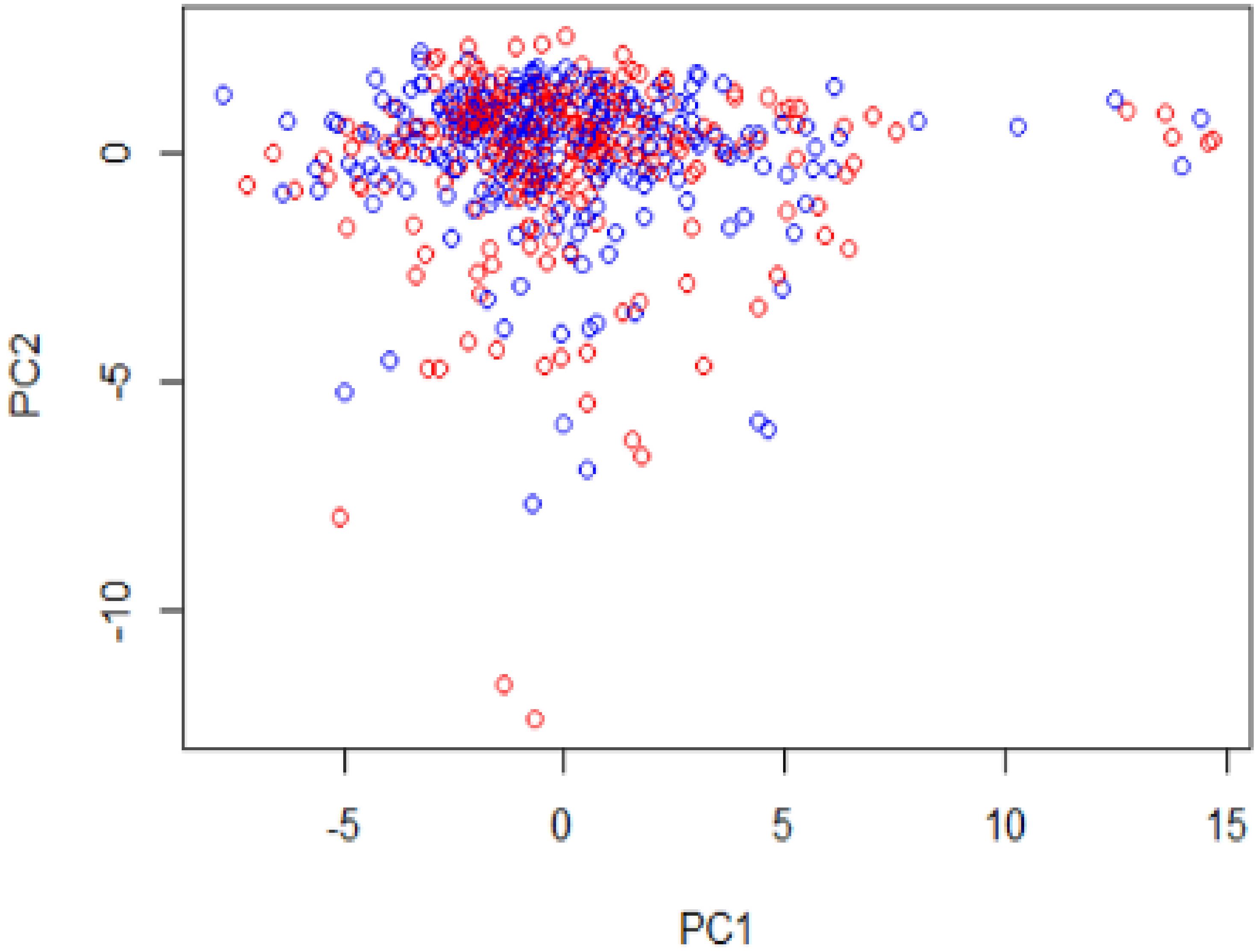

Supplement: Supplementary file 1 — Supplementary Figure 1. [file 41598_2023_47983_MOESM1_ESM.pdf]
