# Supplementary material for: Ovarian recurrence risk assessment using machine learning, clinical information, and serum protein levels to predict survival in high grade ovarian cancer
Source: Sci Rep. 2023 Nov 27;13:20933. doi: 10.1038/s41598-023-47983-z (PMC10684567; doi:10.1038/s41598-023-47983-z)
Supplement: Supplementary file 2 — Supplementary Figure 2. [file 41598_2023_47983_MOESM2_ESM.pdf]

Supplementary Figure 2. Each page shows first the differing variable coefficients followed by the Kaplan Meier curve demonstrating difference in time to recurrence (TTR) between high-risk and low-risk patients at the associated changes in alpha. The changes in alpha range from 0 – 1 with increases by 0.1.

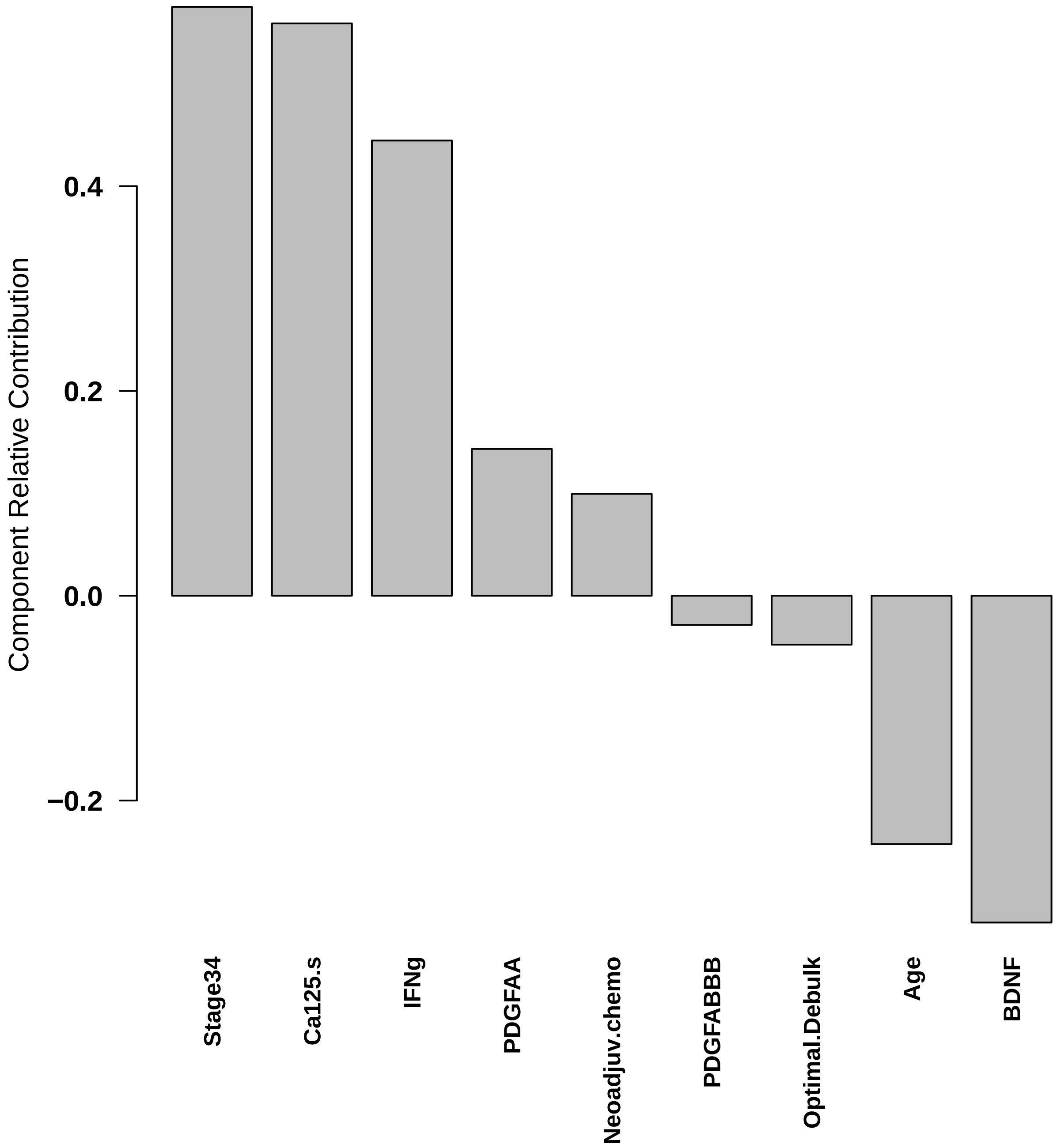

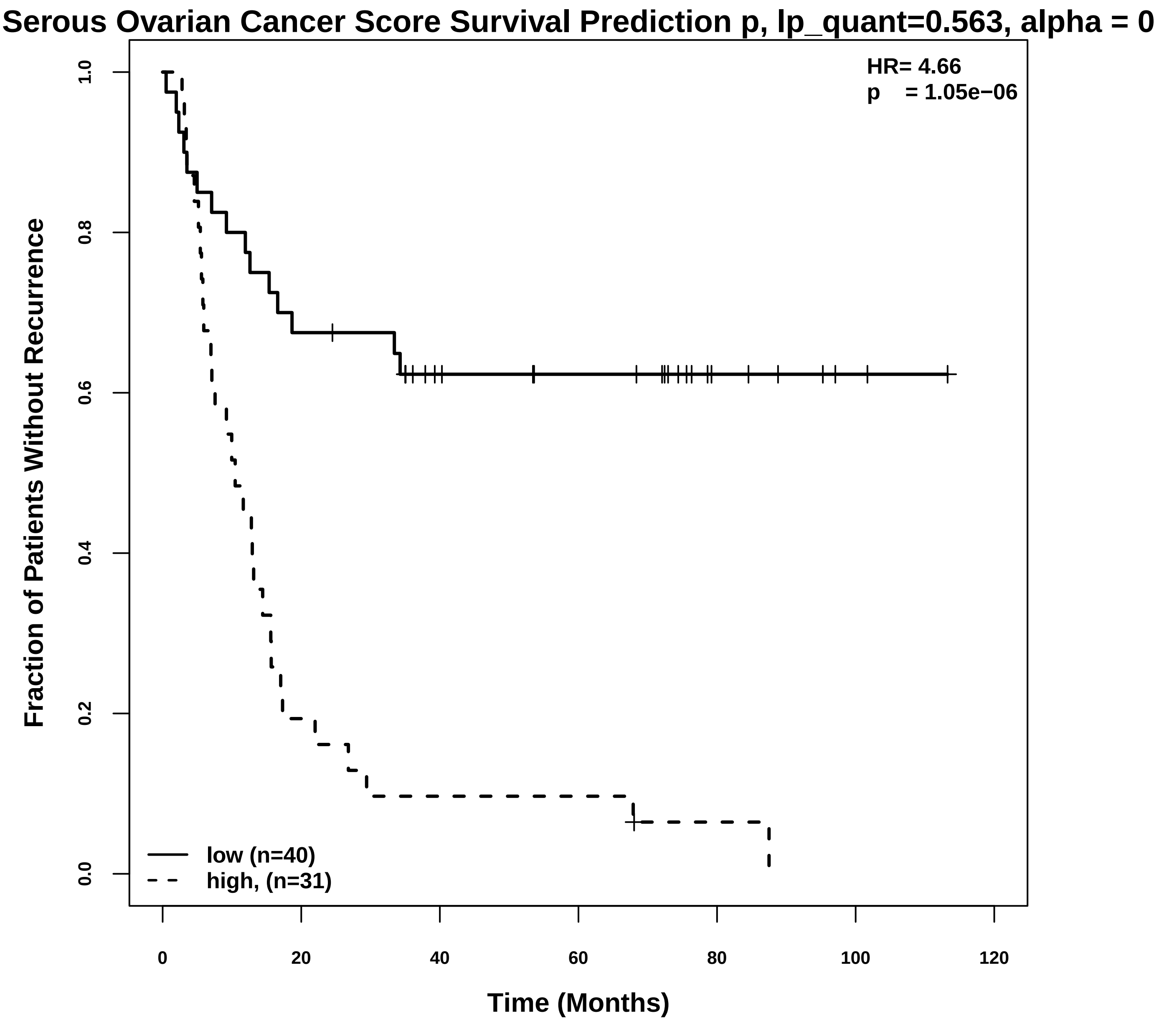

Serous Ovarian Cancer Score Survival Prediction p, lp_quant=0.563, alpha = 0
HR= 4.66
p = 1.05e−06
Fraction of Patients Without Recurrence
0.0
0.2
0.4
0.6
0.8
1.0
0
20
40
60
80
100
120
Time (Months)
low (n=40)
high, (n=31)

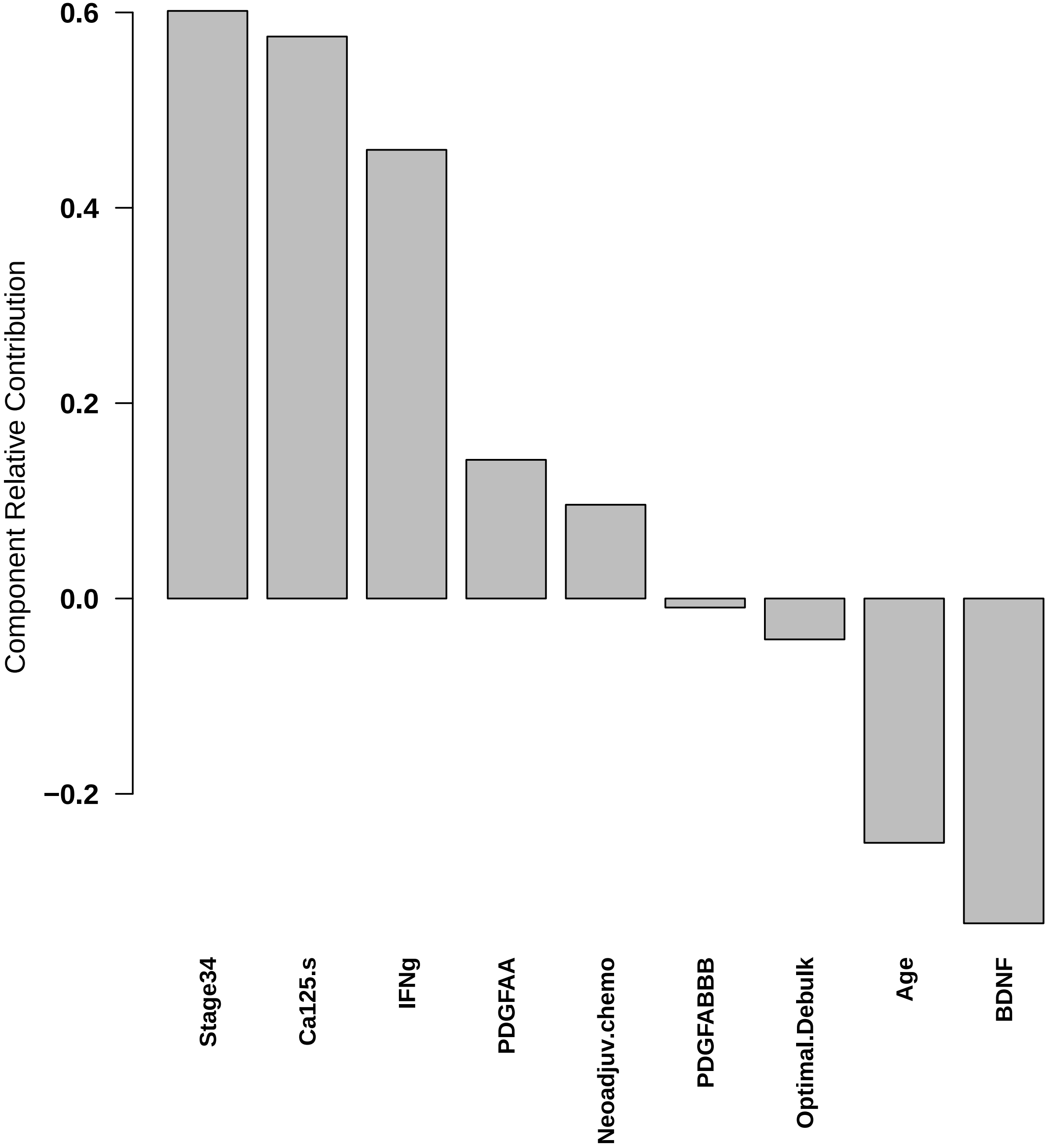

Component Relative Contribution
0.6
0.4
0.2
0.0
−0.2
Stage34
Ca125.s
IFNg
PDGFAA
Neoadjuv.chemo
PDGFABBB
Optimal.Debulk
Age
BDNF

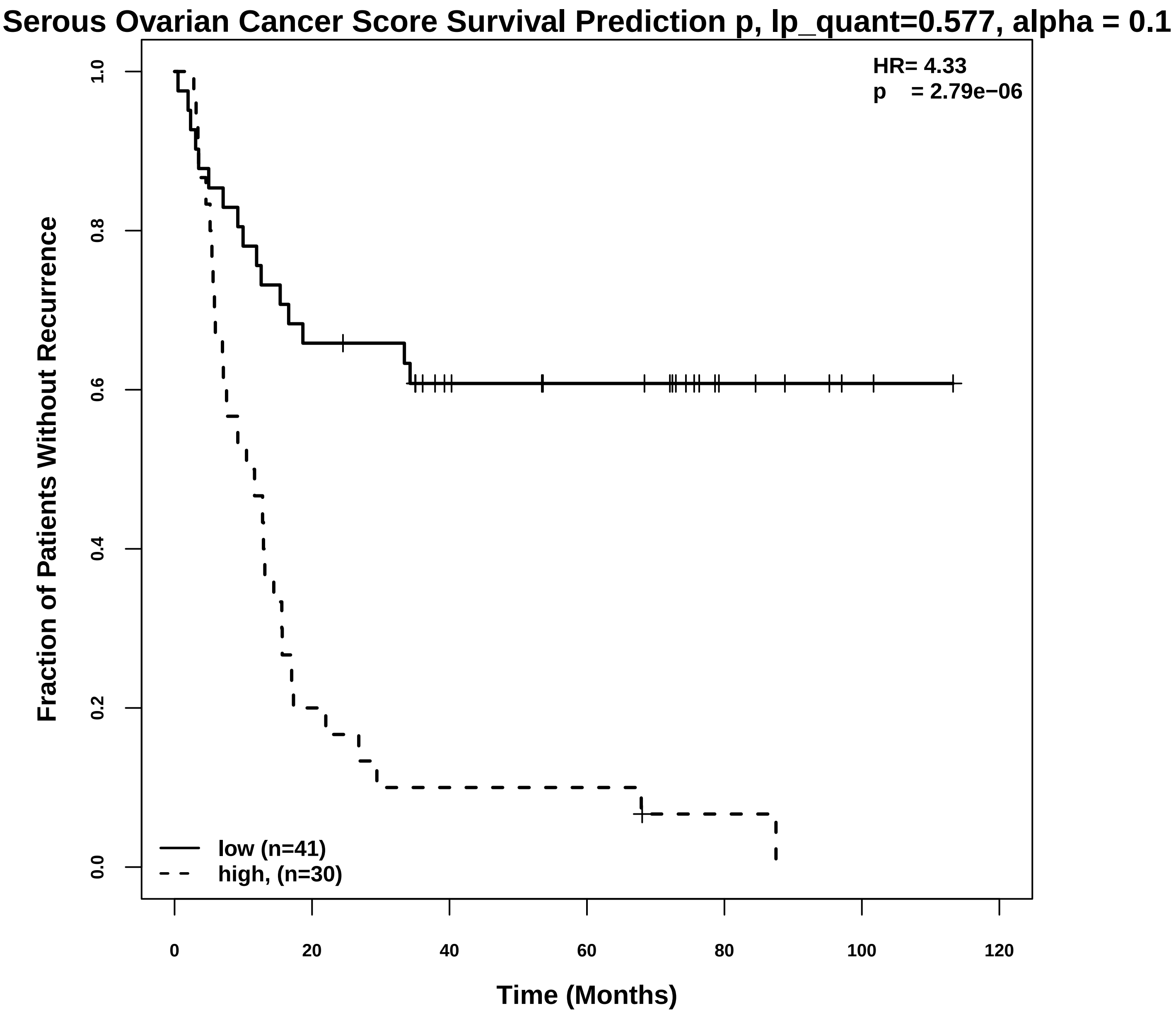
Serous Ovarian Cancer Score Survival Prediction p, lp_quant=0.577, alpha = 0.1
HR= 4.33
p = 2.79e−06
Fraction of Patients Without Recurrence
0.0
0.2
0.4
0.6
0.8
1.0
0
20
40
60
80
100
120
Time (Months)
low (n=41)
high, (n=30)

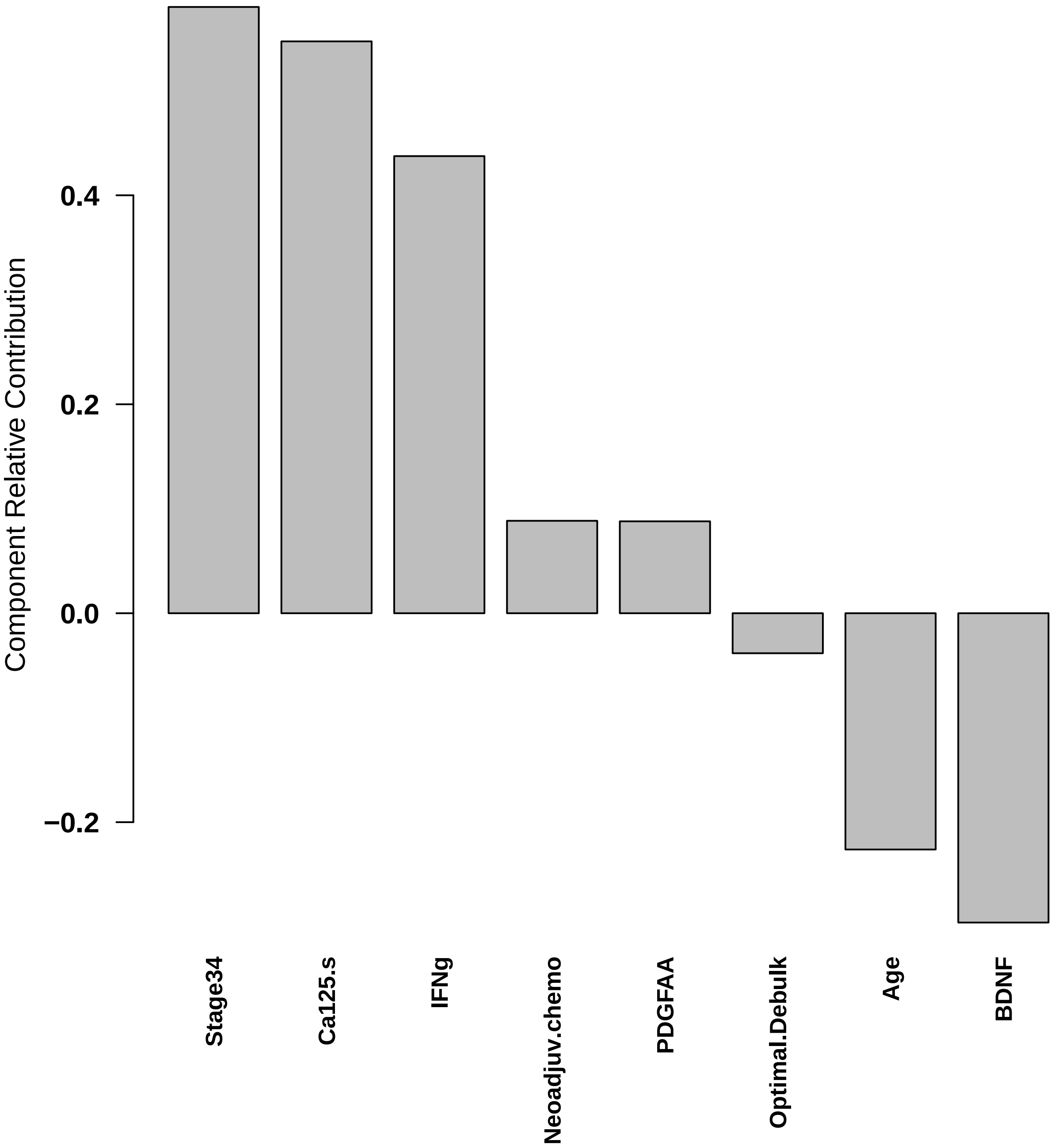

Component Relative Contribution
0.4
0.2
0.0
−0.2
Stage34
Ca125.s
IFNg
Neoadjuv.chemo
PDGFAA
Optimal.Debulk
Age
BDNF

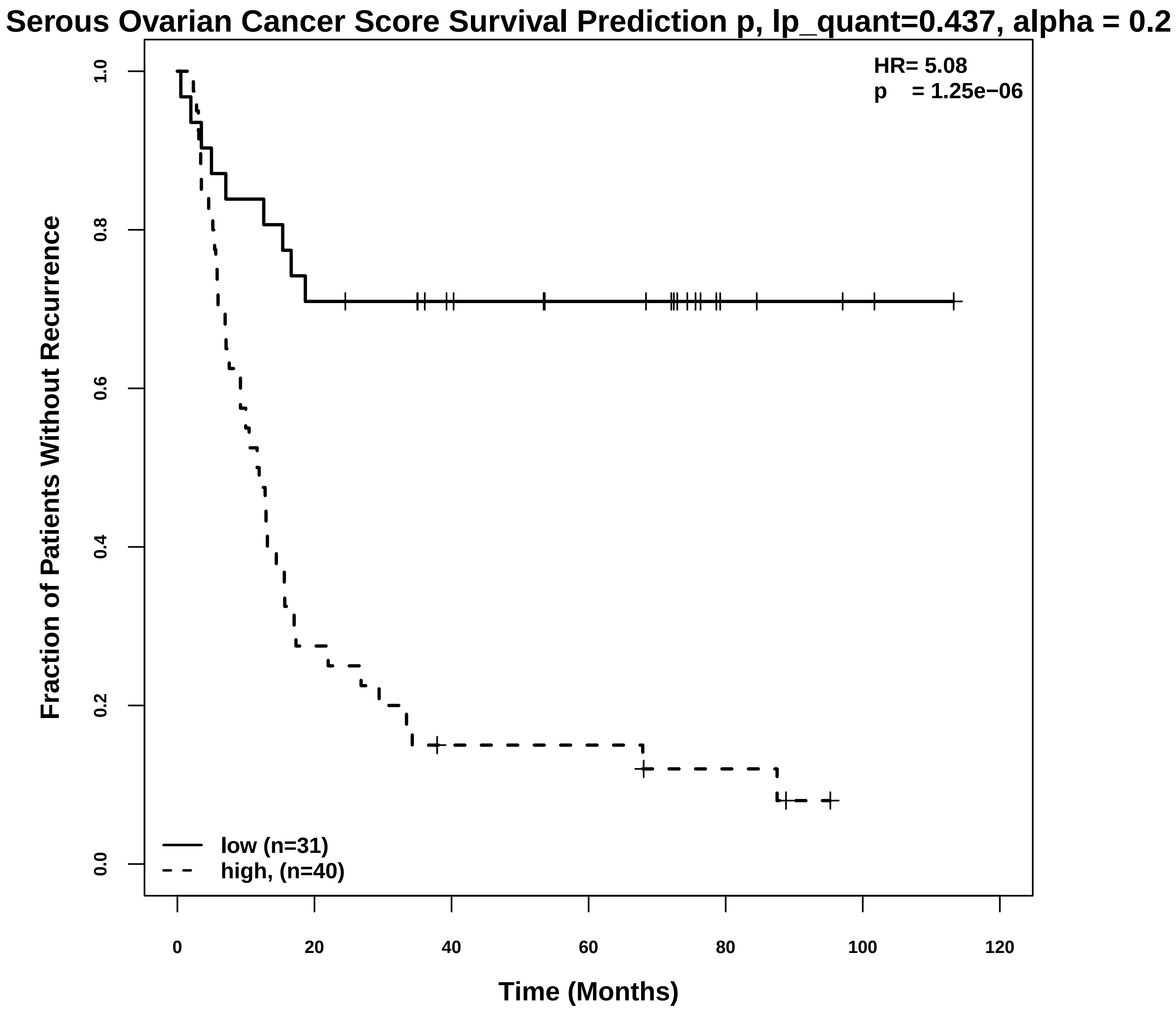
Serous Ovarian Cancer Score Survival Prediction p, lp_quant=0.437, alpha = 0.2
HR= 5.08
p = 1.25e−06
Fraction of Patients Without Recurrence
0.0
0.2
0.4
0.6
0.8
1.0
low (n=31)
high, (n=40)
0
20
40
60
80
100
120
Time (Months)

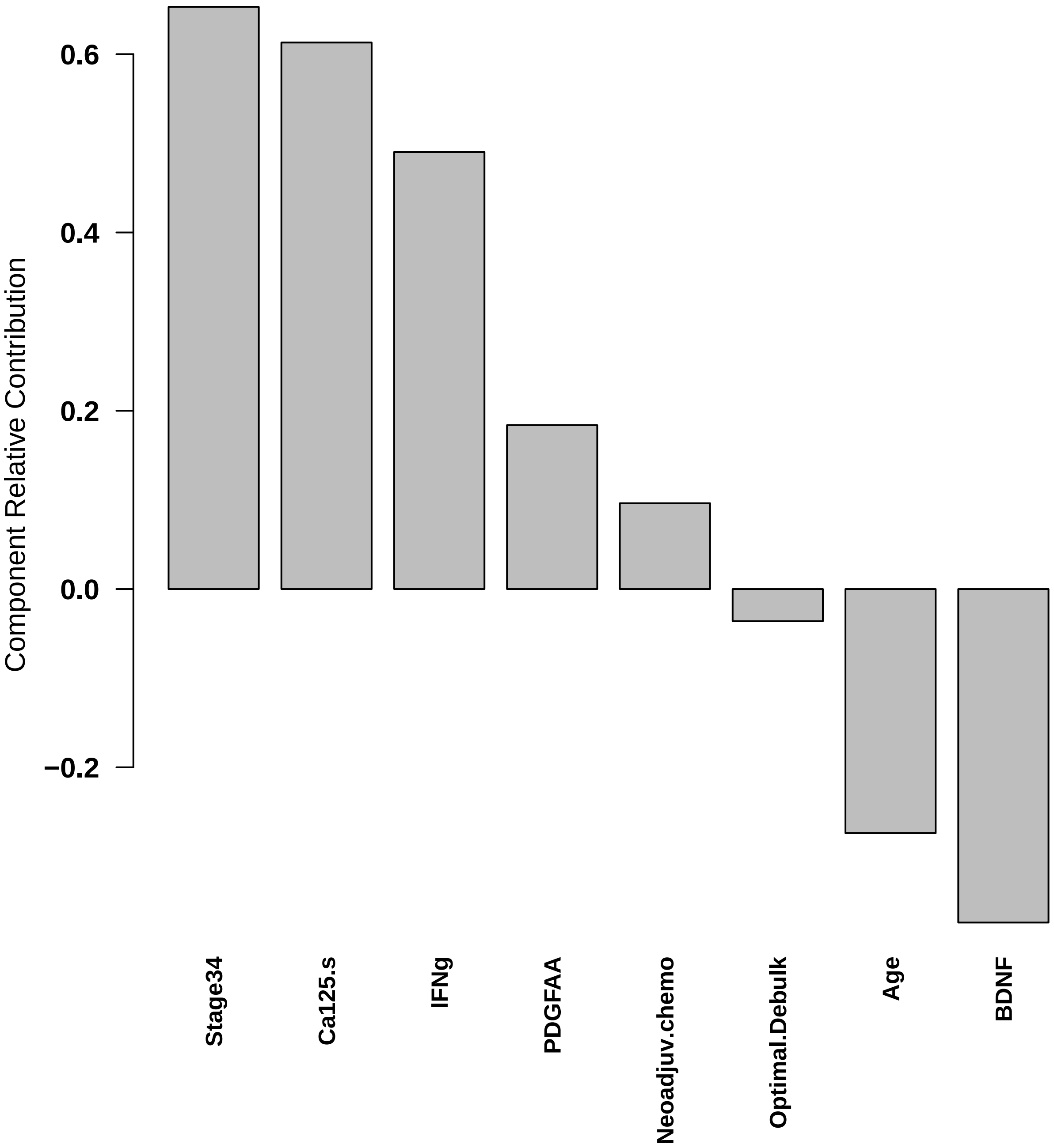

0.6
0.4
0.2
0.0
−0.2
Component Relative Contribution
Stage34
Ca125.s
IFNg
PDGFAA
Neoadjuv.chemo
Optimal.Debulk
Age
BDNF

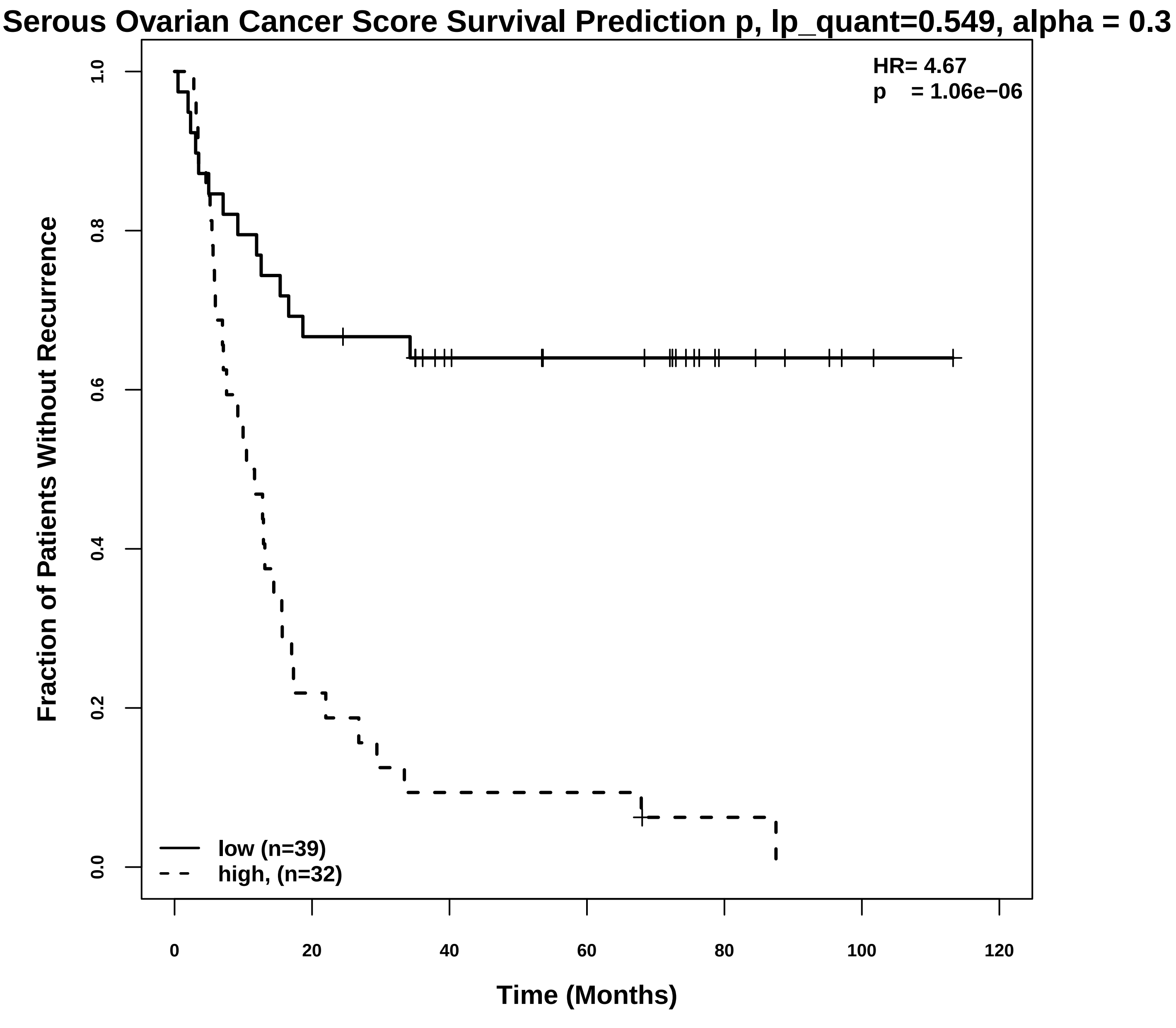

Serous Ovarian Cancer Score Survival Prediction p, lp_quant=0.549, alpha = 0.3
HR= 4.67
p = 1.06e−06
Fraction of Patients Without Recurrence
1.0
0.8
0.6
0.4
0.2
0.0
low (n=39)
high, (n=32)
0
20
40
60
80
100
120
Time (Months)

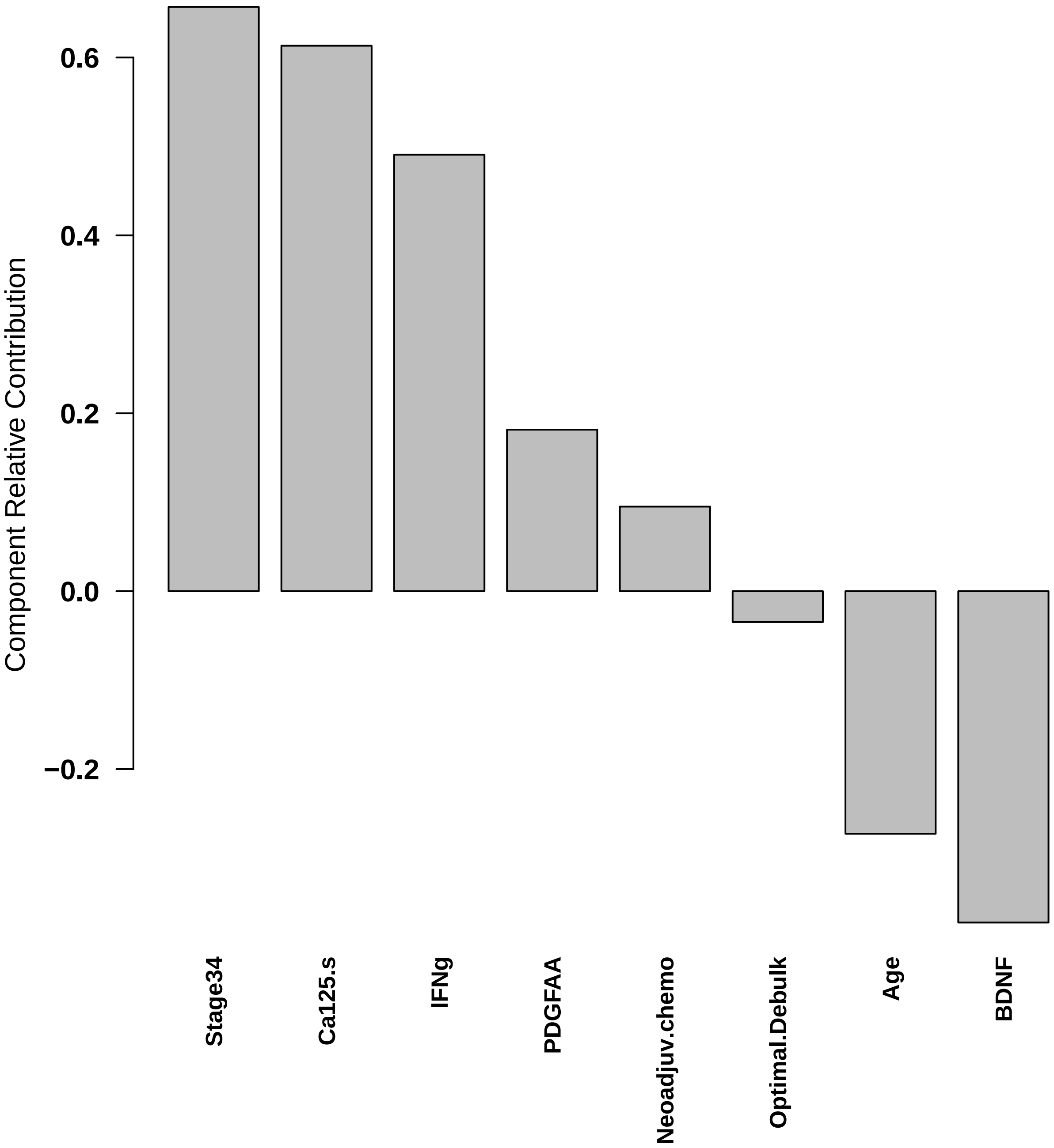

Component Relative Contribution
0.6
0.4
0.2
0.0
−0.2
Stage34
Ca125.s
IFNg
PDGFAA
Neoadjuv.chemo
Optimal.Debulk
Age
BDNF

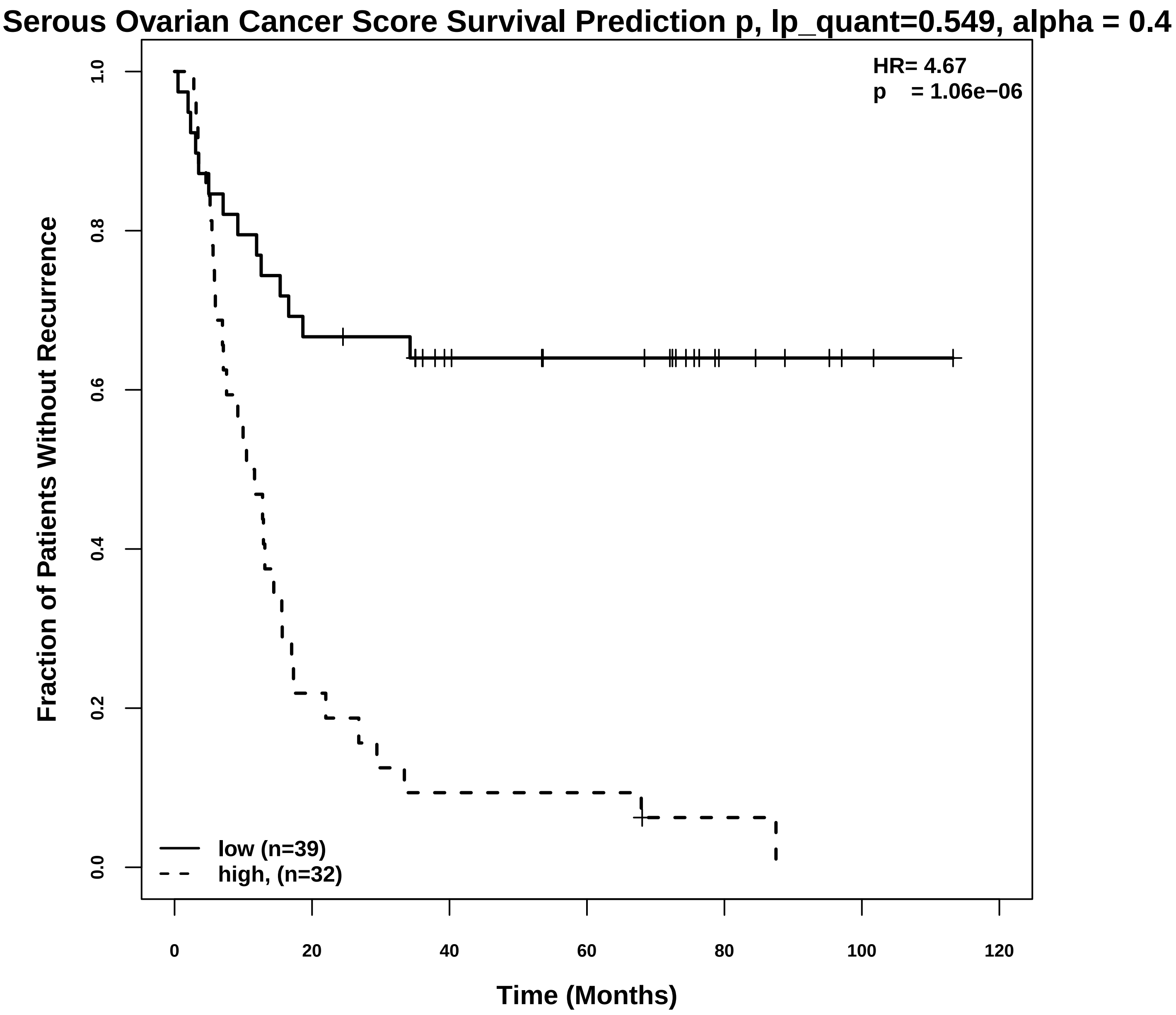

Serous Ovarian Cancer Score Survival Prediction p, lp_quant=0.549, alpha = 0.4
HR= 4.67
p = 1.06e−06
Fraction of Patients Without Recurrence
0.0
0.2
0.4
0.6
0.8
1.0
0
20
40
60
80
100
120
Time (Months)
low (n=39)
high, (n=32)

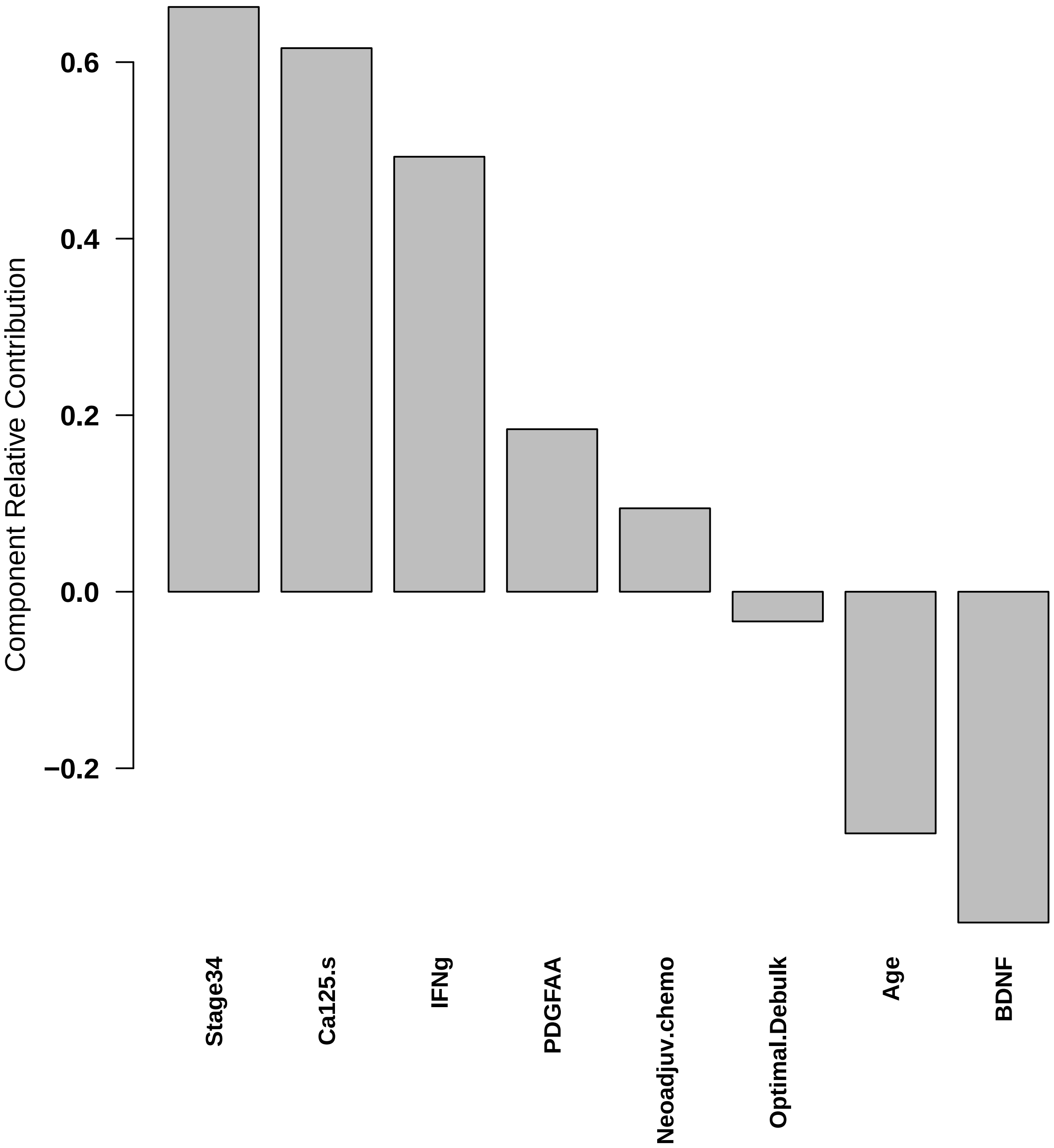

Component Relative Contribution
0.6
0.4
0.2
0.0
−0.2
Stage34
Ca125.s
IFNg
PDGFAA
Neoadjuv.chemo
Optimal.Debulk
Age
BDNF

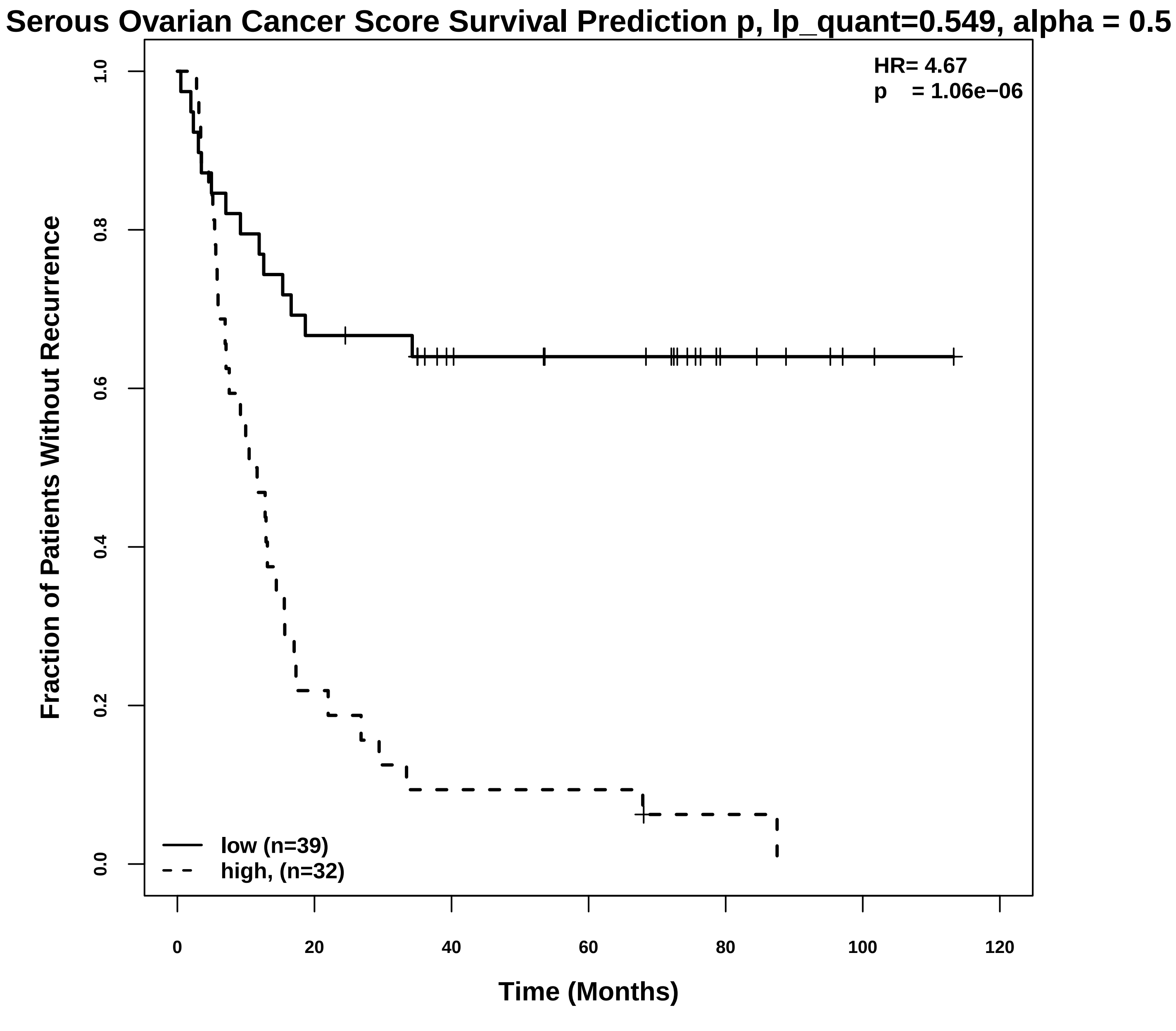

Serous Ovarian Cancer Score Survival Prediction p, lp_quant=0.549, alpha = 0.5
HR= 4.67
p = 1.06e−06
Fraction of Patients Without Recurrence
1.0
0.8
0.6
0.4
0.2
0.0
0
20
40
60
80
100
120
Time (Months)
low (n=39)
high, (n=32)

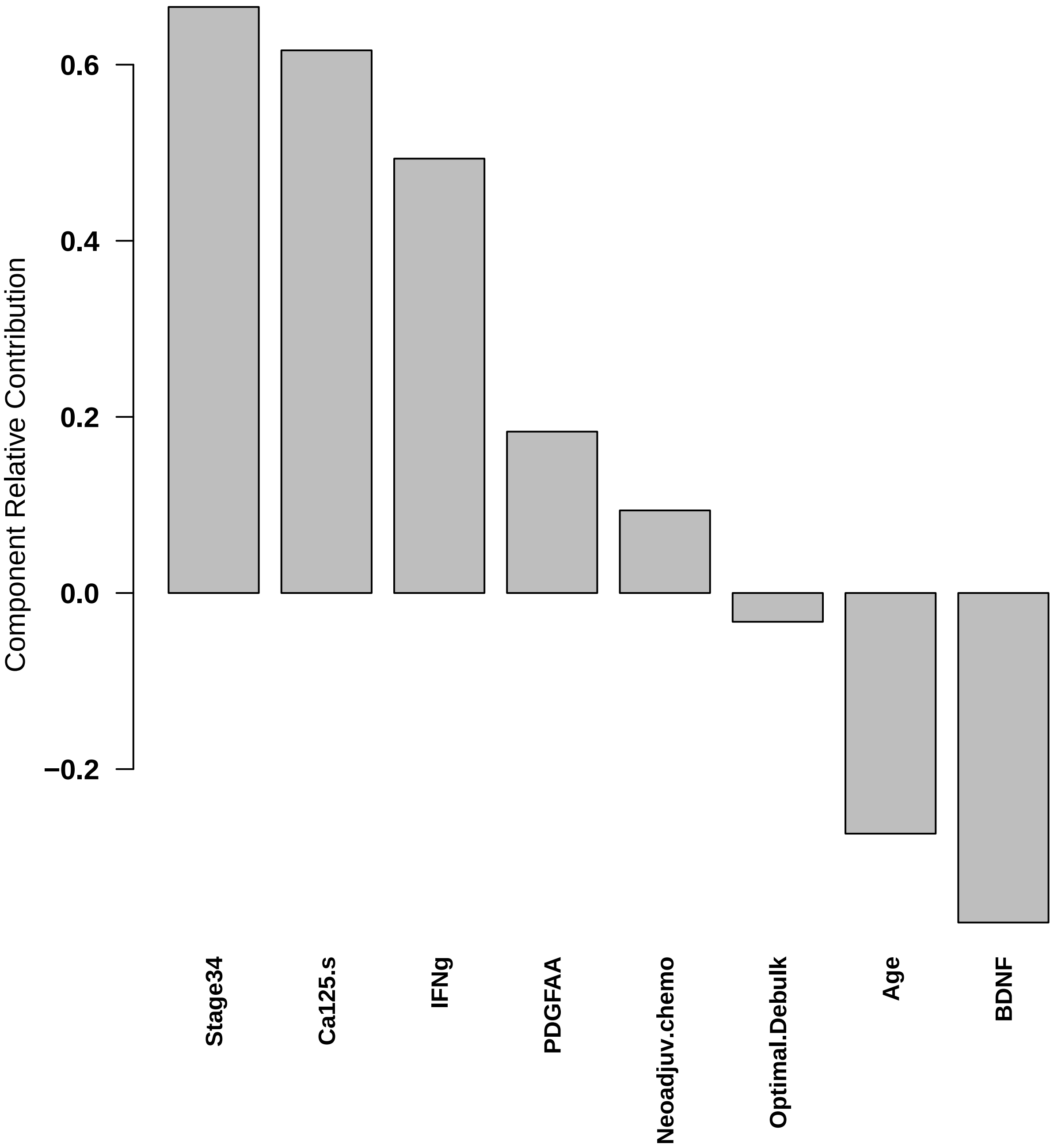

Component Relative Contribution
0.6
0.4
0.2
0.0
−0.2
Stage34
Ca125.s
IFNg
PDGFAA
Neoadjuv.chemo
Optimal.Debulk
Age
BDNF

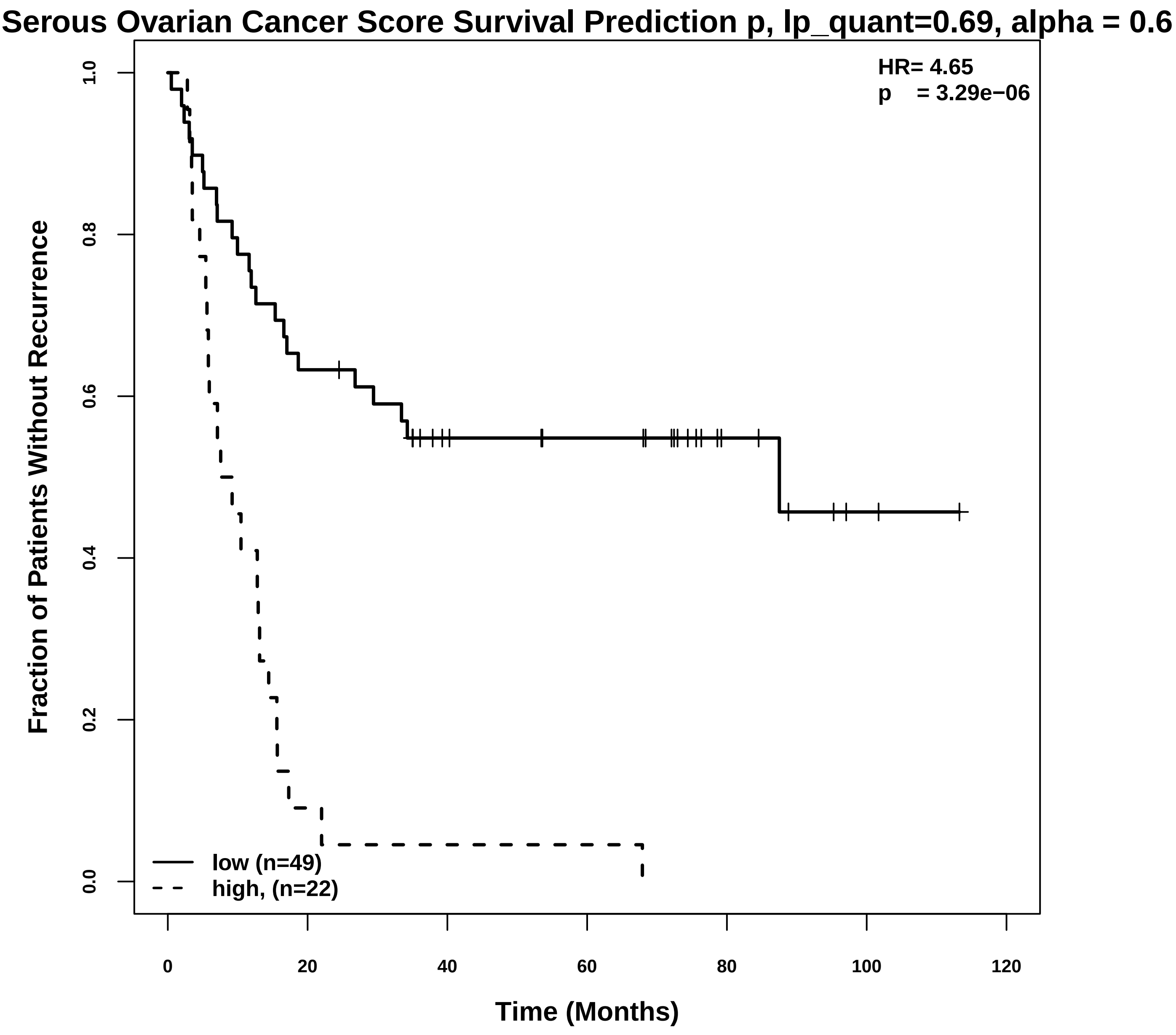
Serous Ovarian Cancer Score Survival Prediction p, lp_quant=0.69, alpha = 0.6
HR= 4.65
p = 3.29e−06
Fraction of Patients Without Recurrence
1.0
0.8
0.6
0.4
0.2
0.0
0
20
40
60
80
100
120
Time (Months)
low (n=49)
high, (n=22)

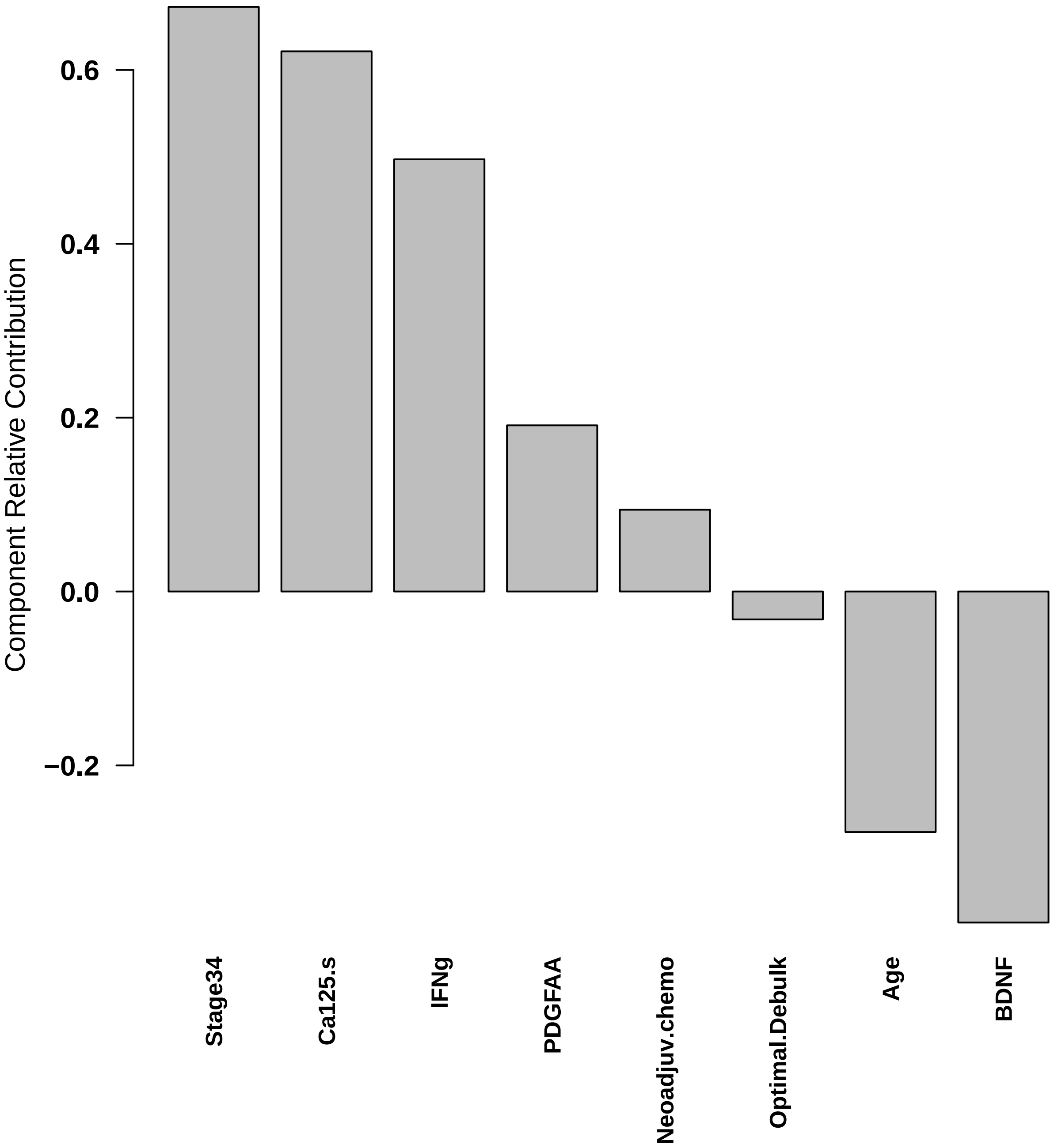

Component Relative Contribution
0.6
0.4
0.2
0.0
−0.2
Stage34
Ca125.s
IFNg
PDGFAA
Neoadjuv.chemo
Optimal.Debulk
Age
BDNF

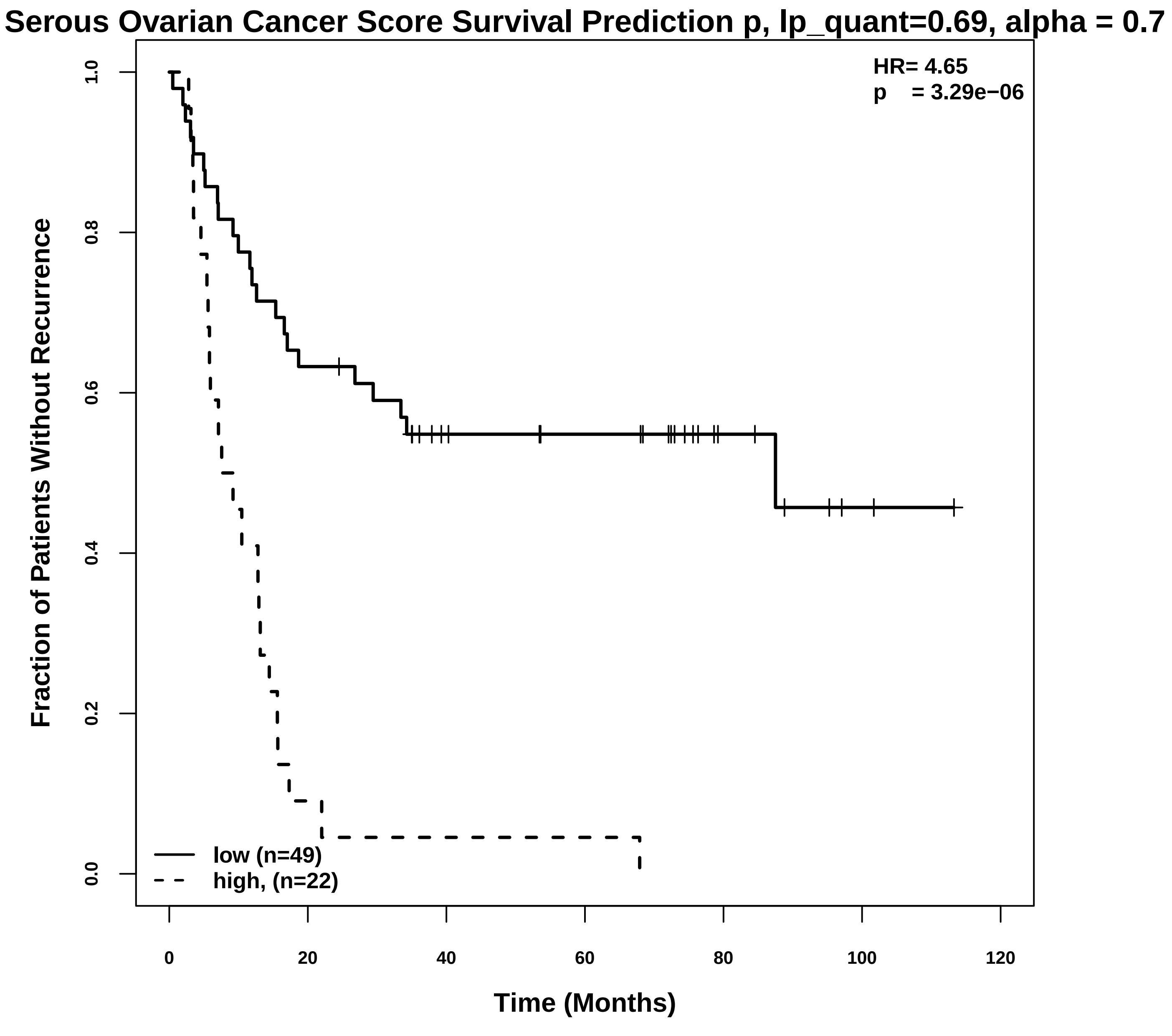

Serous Ovarian Cancer Score Survival Prediction p, lp_quant=0.69, alpha = 0.7
HR= 4.65
p = 3.29e−06
Fraction of Patients Without Recurrence
0.0
0.2
0.4
0.6
0.8
1.0
low (n=49)
high, (n=22)
0
20
40
60
80
100
120
Time (Months)

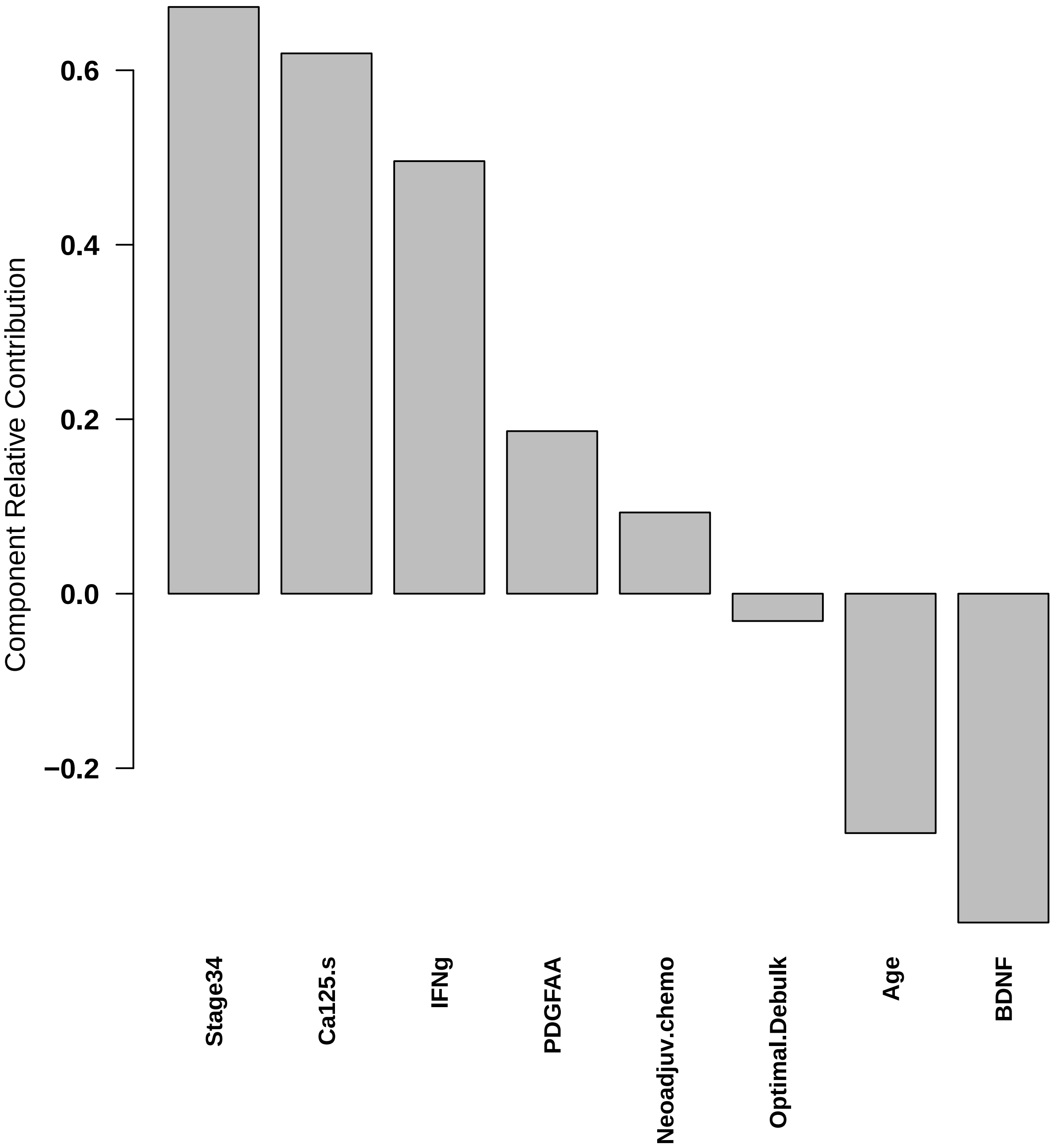

Component Relative Contribution
0.6
0.4
0.2
0.0
−0.2
Stage34
Ca125.s
IFNg
PDGFAA
Neoadjuv.chemo
Optimal.Debulk
Age
BDNF

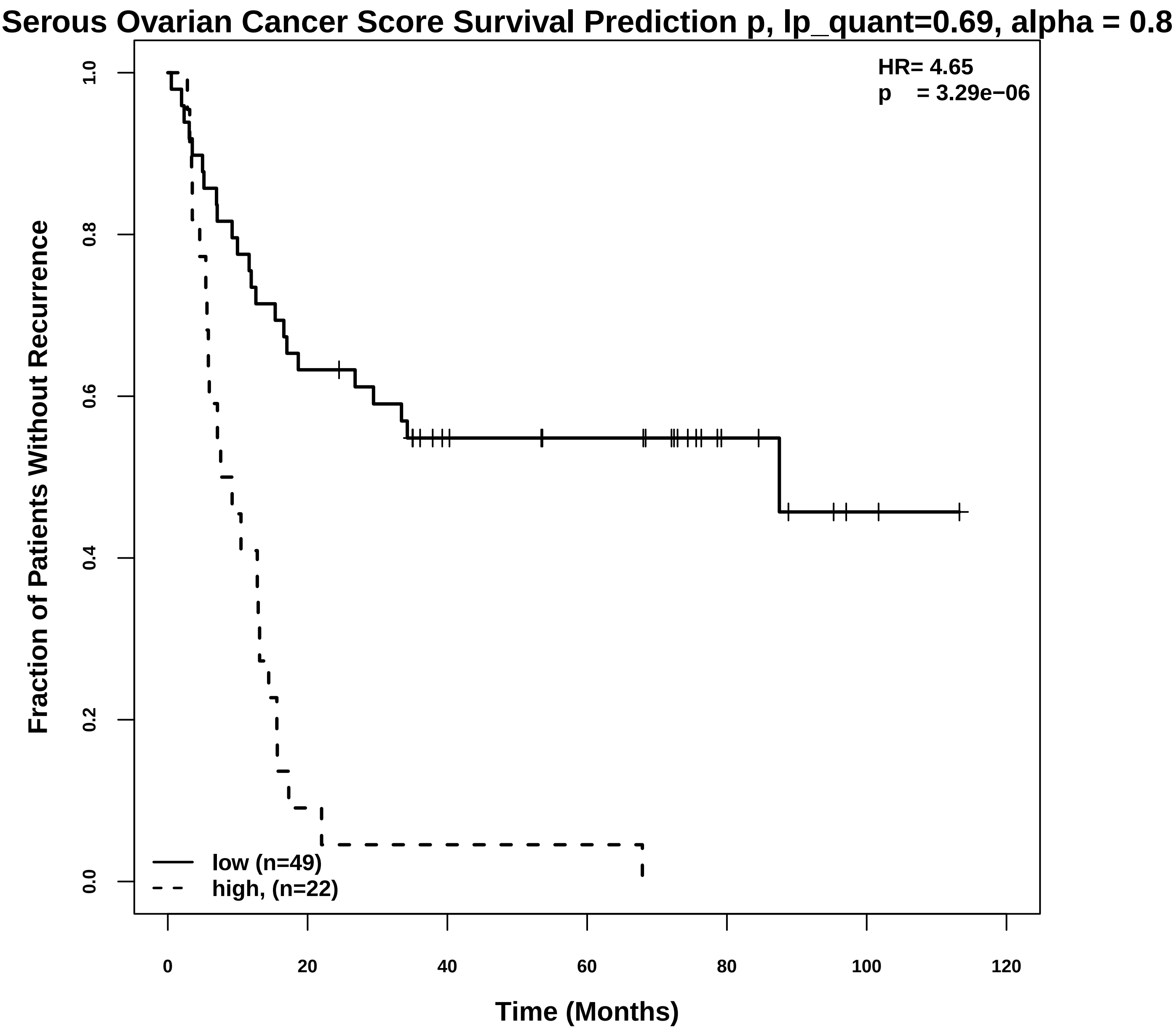
Serous Ovarian Cancer Score Survival Prediction p, lp_quant=0.69, alpha = 0.8
HR= 4.65
p = 3.29e−06
Fraction of Patients Without Recurrence
1.0
0.8
0.6
0.4
0.2
0.0
0
20
40
60
80
100
120
Time (Months)
low (n=49)
high, (n=22)

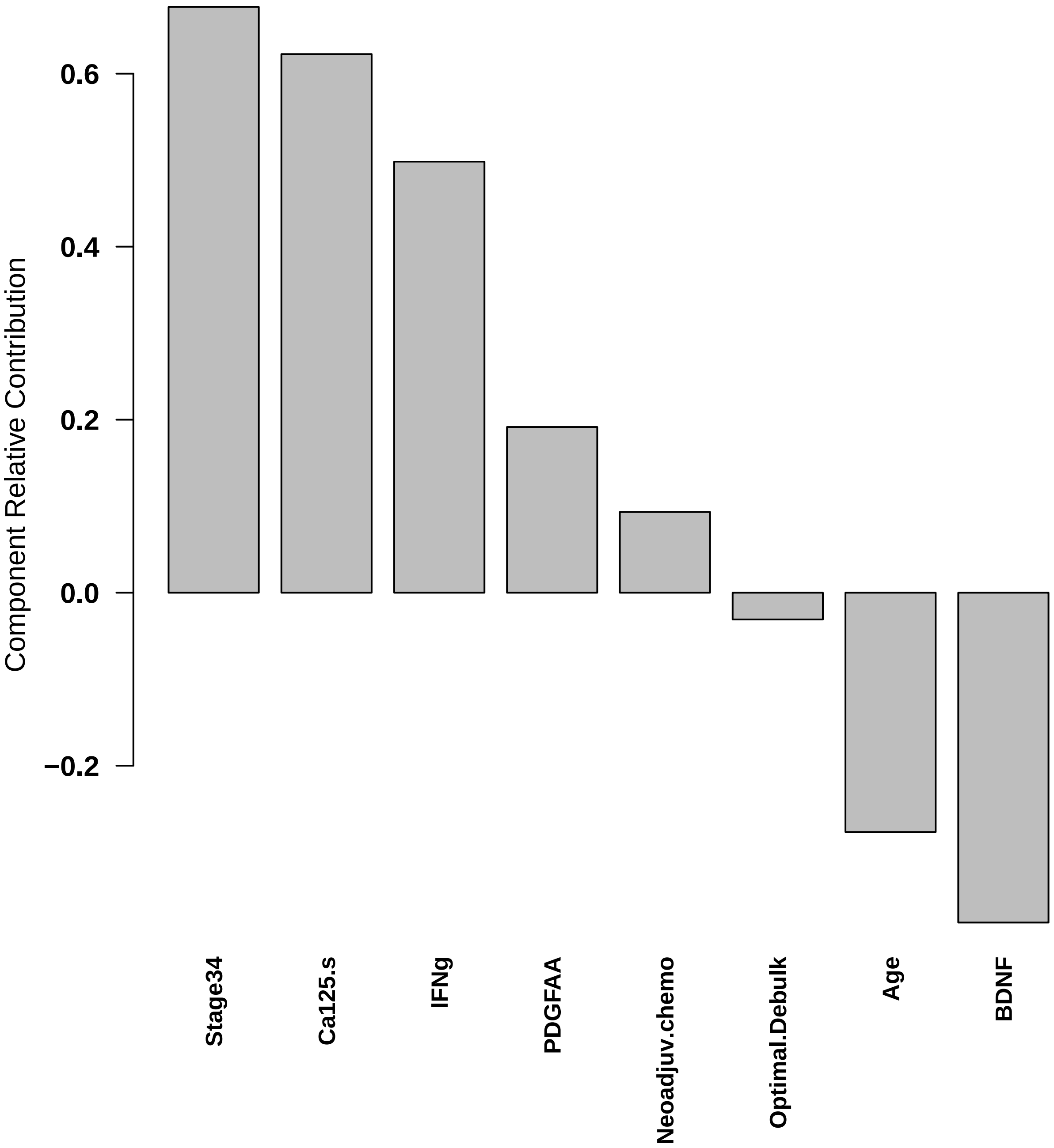

Component Relative Contribution
0.6
0.4
0.2
0.0
−0.2
Stage34
Ca125.s
IFNg
PDGFAA
Neoadjuv.chemo
Optimal.Debulk
Age
BDNF

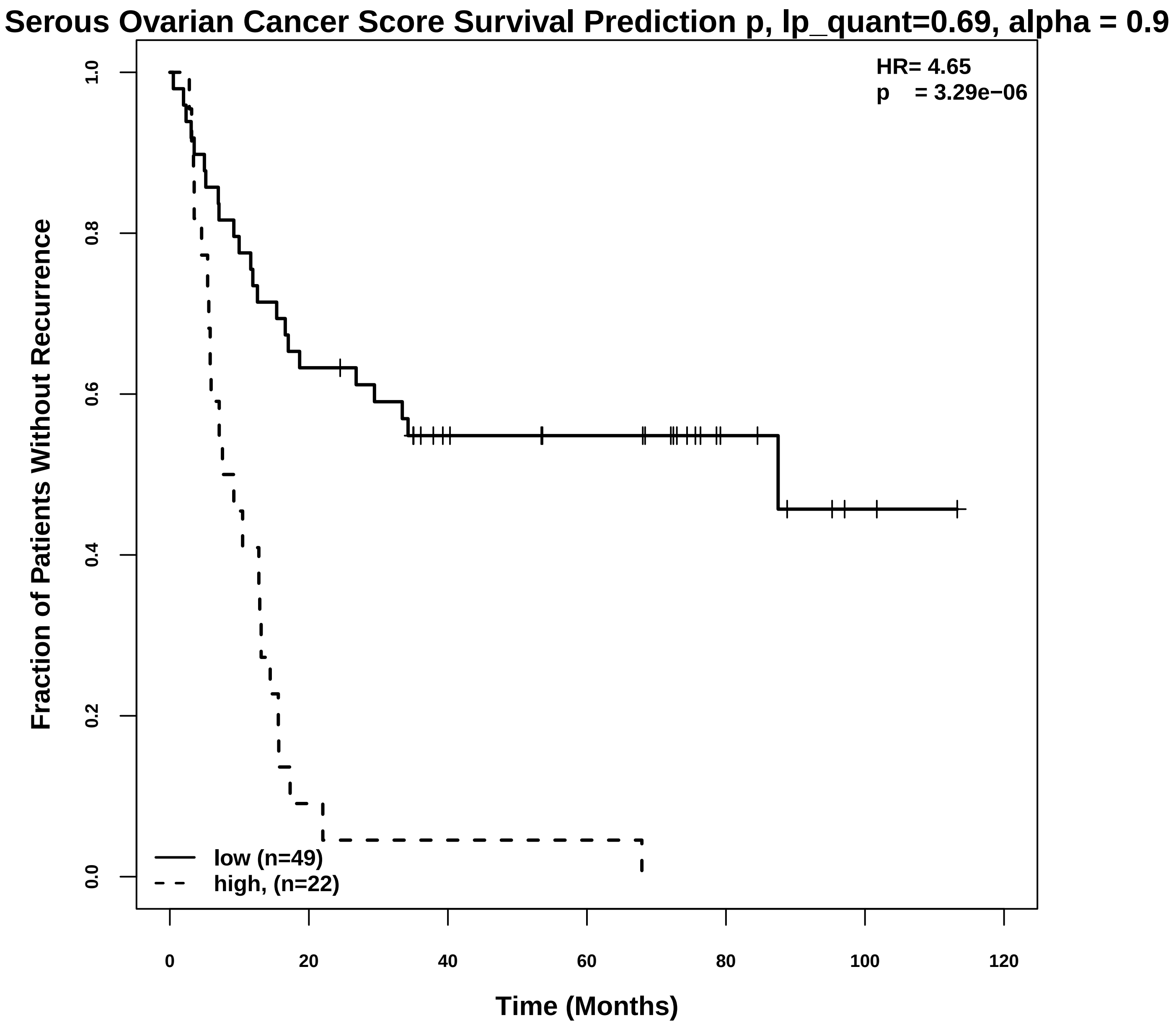

Serous Ovarian Cancer Score Survival Prediction p, lp_quant=0.69, alpha = 0.9
HR= 4.65
p = 3.29e−06
Fraction of Patients Without Recurrence
0.0
0.2
0.4
0.6
0.8
1.0
low (n=49)
high, (n=22)
0
20
40
60
80
100
120
Time (Months)

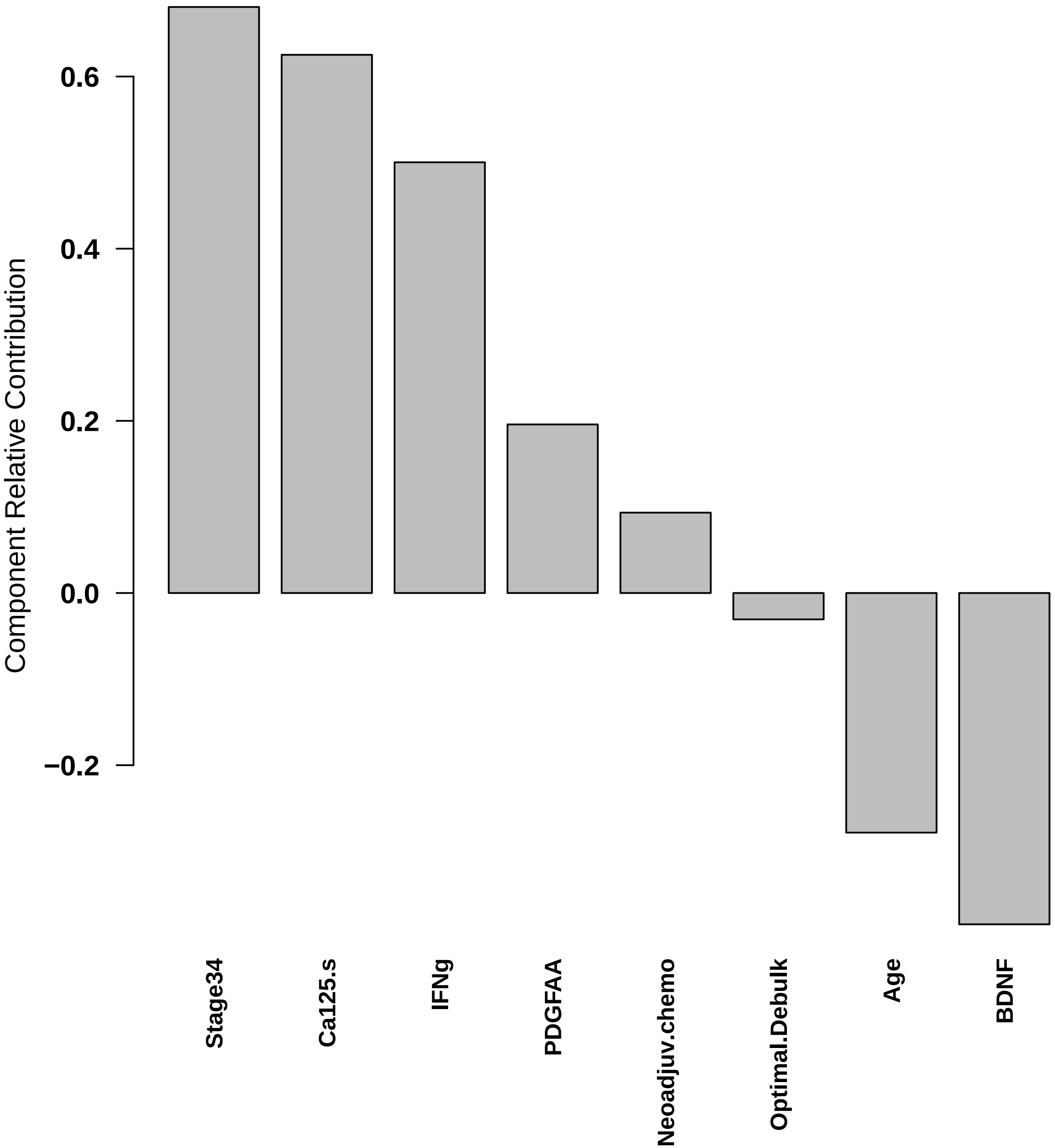

Component Relative Contribution
0.6
0.4
0.2
0.0
−0.2
Stage34
Ca125.s
IFNg
PDGFAA
Neoadjuv.chemo
Optimal.Debulk
Age
BDNF

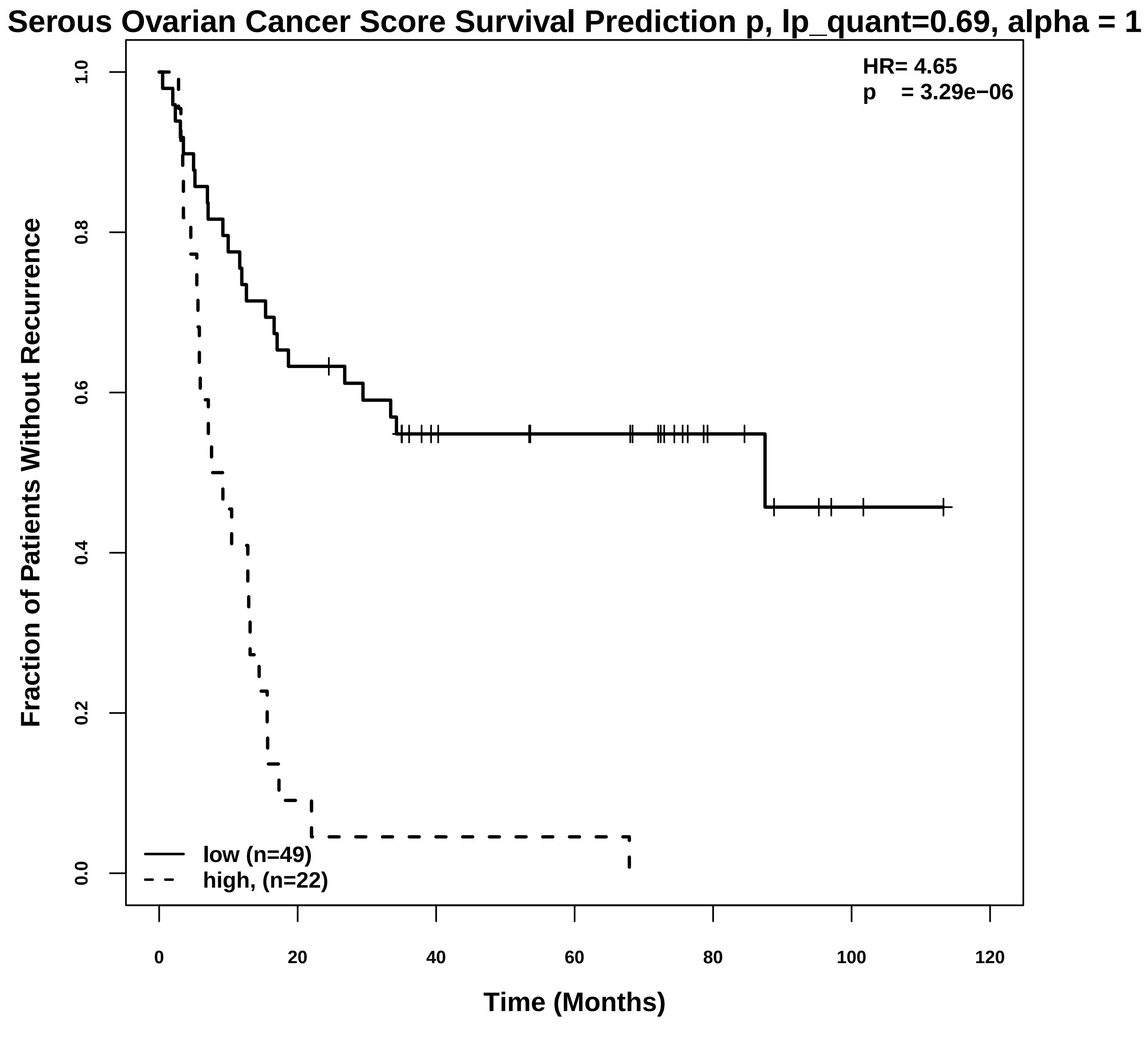
Serous Ovarian Cancer Score Survival Prediction p, lp_quant=0.69, alpha = 1
HR= 4.65
p = 3.29e−06
Fraction of Patients Without Recurrence
1.0
0.8
0.6
0.4
0.2
0.0
0
20
40
60
80
100
120
Time (Months)
low (n=49)
high, (n=22)
