# Supplementary material for: Ovarian recurrence risk assessment using machine learning, clinical information, and serum protein levels to predict survival in high grade ovarian cancer
Source: Sci Rep. 2023 Nov 27;13:20933. doi: 10.1038/s41598-023-47983-z (PMC10684567; doi:10.1038/s41598-023-47983-z)
Supplement: Supplementary file 3 — Supplementary Table 1. [file 41598_2023_47983_MOESM3_ESM.pdf]

Supplementary Table 1. Serum protein survival prediction after harmonization.

Time to Recurrence									Time To Death								
Mol	cutoff	Low.n	High.n	HR	1/HR	CI-L	CI-H	Logrank-p	Mol	cutoff	Low.n	High.n	HR	1/HR	CI-L	CI-H	Logrank-p
BDNF	10	8	63	1.044399	0.957489	0.411789	2.648857	0.927102	BDNF	10	8	63	0.693622	1.441708	0.264281	1.820451	0.454933
BDNF	20	15	56	0.648118	1.542928	0.333817	1.258345	0.196703	BDNF	20	15	56	0.645996	1.547998	0.293911	1.419852	0.273015
BDNF	30	22	49	0.4939	2.024701	0.272387	0.895555	0.017771	BDNF	30	22	49	0.35702	2.80096	0.171989	0.741116	0.003901
BDNF	40	29	42	0.50696	1.972542	0.281564	0.912789	0.021064	BDNF	40	29	42	0.406306	2.461198	0.19135	0.862738	0.015408
BDNF	50	36	35	0.37267	2.683342	0.199848	0.694943	0.001247	BDNF	50	36	35	0.329175	3.037902	0.144818	0.748221	0.005364
BDNF	60	43	28	0.299349	3.340581	0.147381	0.608017	0.000415	BDNF	60	43	28	0.335586	2.979863	0.136308	0.826202	0.012673
BDNF	70	50	21	0.143731	6.957449	0.051215	0.403372	2.01E-05	BDNF	70	50	21	0.202716	4.933018	0.061202	0.671442	0.003811
BDNF	80	57	14	0.113346	8.822573	0.027338	0.469936	0.000303	BDNF	80	57	14	0.118002	8.474438	0.016041	0.868049	0.011723
BDNF	90	64	7	0.315292	3.171661	0.076281	1.303192	0.092196	BDNF	90	64	7	0.298951	3.345028	0.040649	2.1986	0.207882
IFNg	10	8	63	0.7592	1.317176	0.298976	1.927861	0.56108	IFNg	10	8	63	0.597994	1.672256	0.207559	1.722874	0.335629
IFNg	20	15	56	0.590583	1.693241	0.297301	1.173182	0.128253	IFNg	20	15	56	0.49293	2.028686	0.22421	1.083716	0.072388
IFNg	30	22	49	1.16931	0.855205	0.601458	2.273287	0.644369	IFNg	30	22	49	0.859202	1.16387	0.390691	1.889543	0.705607
IFNg	40	29	42	1.598389	0.62563	0.858589	2.975634	0.135569	IFNg	40	29	42	1.345993	0.742946	0.62466	2.900292	0.446443
IFNg	50	36	35	1.261374	0.792786	0.700834	2.270246	0.437683	IFNg	50	36	35	1.039163	0.962313	0.501115	2.154913	0.917772
IFNg	60	43	28	1.396853	0.715895	0.771132	2.530301	0.268038	IFNg	60	43	28	1.345301	0.743328	0.646775	2.798242	0.425623
IFNg	70	50	21	1.877229	0.5327	1.01484	3.472457	0.041443	IFNg	70	50	21	1.510319	0.662112	0.701628	3.251101	0.288454
IFNg	80	57	14	2.138061	0.467714	1.076615	4.245997	0.02629	IFNg	80	57	14	1.669513	0.598977	0.712247	3.913351	0.233215
IFNg	90	64	7	5.462794	0.183057	2.259621	13.20669	2.47E-05	IFNg	90	64	7	2.66851	0.374741	1.015127	7.014833	0.038374
PDGFAA	10	8	63	0.837993	1.193327	0.3545	1.98091	0.686809	PDGFAA	10	8	63	0.667235	1.498722	0.254133	1.751851	0.408159
PDGFAA	20	15	56	0.668413	1.496082	0.344408	1.297229	0.230643	PDGFAA	20	15	56	0.804761	1.242604	0.355883	1.819813	0.601133
PDGFAA	30	22	49	0.679176	1.472372	0.371197	1.242684	0.206649	PDGFAA	30	22	49	0.732795	1.364637	0.349525	1.536339	0.408586
PDGFAA	40	29	42	0.50523	1.979295	0.280282	0.910717	0.020671	PDGFAA	40	29	42	0.591148	1.691624	0.283356	1.233273	0.156507
PDGFAA	50	36	35	0.37364	2.676373	0.199885	0.698436	0.001358	PDGFAA	50	36	35	0.35142	2.845599	0.155146	0.795996	0.008797
PDGFAA	60	43	28	0.31058	3.219778	0.153088	0.630096	0.000629	PDGFAA	60	43	28	0.264108	3.786326	0.100344	0.695144	0.003806
PDGFAA	70	50	21	0.410745	2.434598	0.190396	0.886112	0.019175	PDGFAA	70	50	21	0.388083	2.576771	0.13494	1.116114	0.068432
PDGFAA	80	57	14	0.298922	3.345351	0.10656	0.838539	0.014943	PDGFAA	80	57	14	0.143339	6.976473	0.019493	1.05403	0.026262
PDGFAA	90	64	7	0.348111	2.872648	0.084128	1.440439	0.127366	PDGFAA	90	64	7	0.35357	2.82829	0.048072	2.600528	0.285613
PDGFABBB	10	8	63	0.731645	1.366783	0.325034	1.646918	0.44859	PDGFABBI	10	8	63	0.701669	1.425173	0.267343	1.841603	0.469438
PDGFABBB	20	15	56	0.808927	1.236206	0.417314	1.568034	0.529288	PDGFABBI	20	15	56	0.912925	1.09538	0.389343	2.140611	0.833982
PDGFABBB	30	22	49	0.713739	1.401073	0.389998	1.306221	0.271872	PDGFABBI	30	22	49	1.074492	0.930672	0.48888	2.361591	0.858047
PDGFABBB	40	29	42	0.589178	1.697281	0.327503	1.059929	0.074129	PDGFABBI	40	29	42	0.688753	1.4519	0.331941	1.429109	0.313952
PDGFABBB	50	36	35	0.397107	2.518212	0.212493	0.742114	0.002761	PDGFABBI	50	36	35	0.394415	2.535397	0.178982	0.869157	0.01686
PDGFABBB	60	43	28	0.300081	3.332433	0.147752	0.609457	0.000426	PDGFABBI	60	43	28	0.347702	2.876024	0.141293	0.855645	0.016125
PDGFABBB	70	50	21	0.237586	4.20901	0.100023	0.564341	0.000414	PDGFABBI	70	50	21	0.154552	6.470324	0.036708	0.650709	0.003396
PDGFABBB	80	57	14	0.270915	3.691201	0.096596	0.759808	0.007873	PDGFABBI	80	57	14	0.121404	8.236971	0.016502	0.893162	0.013323
PDGFABBB	90	64	7	0.369955	2.703033	0.089389	1.53113	0.15297	PDGFABBI	90	64	7	0.418728	2.388186	0.056865	3.083304	0.37789
